# New PPARα Agonist A190-Loaded Microemulsion for Chemotherapy-Induced Peripheral Neuropathy

**DOI:** 10.1021/acs.molpharmaceut.4c01374

**Published:** 2025-01-29

**Authors:** Rudra Pangeni, Surendra Poudel, Sara M. Herz, Grant Berkbigler, Adam S. Duerfeldt, M. Imad Damaj, Qingguo Xu

**Affiliations:** † Department of Pharmaceutics, School of Pharmacy, Virginia Commonwealth University, Richmond, Virginia 23298, United States; ‡ Department of Pharmacology and Toxicology, School of Medicine, Virginia Commonwealth University, Richmond, Virginia 23298, United States; § Department of Medicinal Chemistry, College of Pharmacy, University of Minnesota, Minneapolis, Minnesota 55455, United States; ∥ Departments of Ophthalmology, Pediatrics, Biomedical Engineering, and Massey Cancer Center, Center for Pharmaceutical Engineering, and Center for Drug Discovery, Virginia Commonwealth University, Richmond, Virginia 23298, United States

**Keywords:** peroxisome proliferator-activated receptor, nonaddictive analgesic, oral bioavailability, permeability, peripheral neuropathy, chronic inflammatory pain

## Abstract

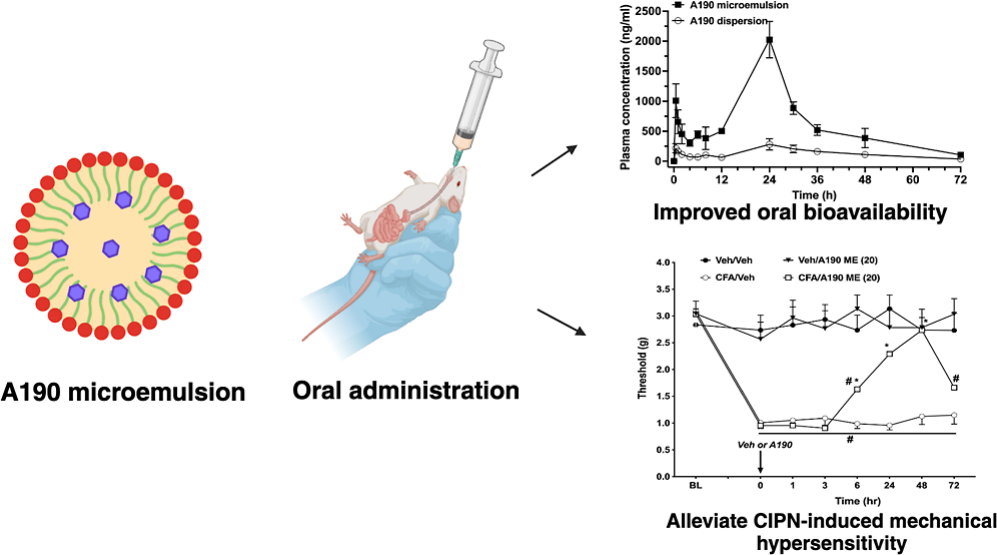

Chemotherapy-induced peripheral neuropathy (CIPN) is a serious side effect of anticancer agents with limited effective preventive or therapeutic interventions. Although fenofibrate, a peroxisome proliferator-activated receptor-alpha (PPARα) agonist, has demonstrated neuroprotective and analgesic properties, its clinical utility is hindered by low receptor affinity, poor subtype selectivity, and suboptimal bioavailability. A190, a highly selective and potent nonfibrate PPARα agonist, offers a promising alternative but is limited by poor aqueous solubility, resulting in reduced oral bioavailability and therapeutic efficacy. To address these limitations, an optimized oil-in-water (o/w) microemulsion formulation was developed using Box–Behnken design to enhance the solubility and intestinal permeability of A190. The A190 microemulsion exhibited physical stability with a droplet size of approximately 100 nm and a drug loading efficiency of greater than 95%. The effective and apparent permeability of A190 from the microemulsion was significantly higher compared to that of free A190 dispersion, respectively. Additionally, no significant impact on the cell viability was observed, indicating less toxicity and a good biocompatibility of the formulation components. The oral bioavailability of A190 microemulsion was approximately 5-fold higher compared to A190 dispersion, demonstrating the microemulsion’s potential to greatly enhance the oral bioavailability of hydrophobic drugs. Furthermore, our findings reveal that orally administered A190 microemulsion effectively reduced CIPN-induced mechanical hypersensitivity, likely mediated through PPARα activation. A190 microemulsion was found to be equally effective at reducing the chronic inflammatory complete Freund’s adjuvant-induced pain. These results underscore A190s potential as a nonopioid therapeutic candidate, utilizing a novel microemulsion formulation for the management of chemotherapy-induced neuropathic pain and chronic inflammatory pain.

## 1 Introduction

Chemotherapy-induced peripheral neuropathy (CIPN) is a serious potential side effect of anticancer drugs, including taxanes- and platinum-based compounds, vinca alkaloids, epothilones, and bortezomib.^1^ CIPN often begins as acute sensory pain and may progress into chronic neuropathy with repeated treatment cycles.^2^ The pathophysiology of CIPN is highly complex, involving multiple mechanisms specific to each anticancer drug class, which complicates its treatment and prevention.^3^ Presently, there are no approved or effective preventive agents for the CIPN, and several previous clinical trials involving antiepileptic and antidepressant drugs have shown mixed results. While duloxetine has shown moderate benefits in managing painful CIPN in a randomized controlled trial, other pharmacological agents and nutraceuticals lack endorsement per 2020 American Cancer Society (ACS) and American Society of Clinical Oncology (ASCO) guidelines, which recommend tapering duloxetine to avoid withdrawal symptoms.^2,4^ Studies have explored drug classes like tricyclic antidepressants, dual serotonin and norepinephrine reuptake inhibitors (SNRIs), anticonvulsants, and opioid agonists, but these drugs exhibit an unfavorable risk/benefit ratio and cause withdrawal-like symptoms.^5^ Following the first report by Devchand et al. in 1996, which indicated an increased inflammatory response in peroxisome proliferator-activated receptor-α (PPARα) deficient mice, a large number of studies have investigated and highlighted the significant role of PPARs in reducing peripheral neuropathic pain.^6^ PPARs, a family of nuclear receptors including PPARα, PPAR-β/δ, and PPAR-γ isoforms, are expressed in various tissues, including the pain neuroaxis.^7^ Exogenous agonists of PPARα, such as fenofibrate, have demonstrated analgesic and neuroprotective activity in rodent models of neuropathic pain and inflammation.^8^ Additionally, several glitazones, which exhibit high affinity to PPAR-γ, have also been highlighted for their role in alleviating peripheral neuropathic pain.^9^ Our group recently reported that fenofibrate effectively inhibits the onset of neuropathy induced by paclitaxel.^10^ Earlier studies demonstrated that gabapentin prevented taxol-induced neuropathic pain at a dose of 100 mg/kg i.p., daily injected for 8 days in ICR mice.^11^ In future studies, we plan to include gabapentin as positive control for direct comparison.

Fenofibrate is a U.S. Food and Drug Administration (FDA)-approved hyperlipidemia drug, rapidly hydrolyzed by esterase to the active metabolite, fenofibric acid, which is then transported to the tissues expressing PPARα.^12^ However, fenofibric acid faces several challenges, including low affinity for PPARα, poor selectivity among PPAR subtypes, dose-limiting toxicities, and limited bioavailability.^13^ The low antinociceptive effect of fenofibrate and fenofibric acid might be attributed to their low affinity for PPARα, which also explains why higher doses were required compared to other PPARα agonists such as GW7647, Wy-14643, and palmitoylethanolamide.^14^ Pemafibrate, which received its first global approval in Japan for the treatment of hyperlipidemia (including familial hyperlipidemia), has been shown to be generally safe and well-tolerated, with clinical trials reporting a similar incidence of adverse events between pemafibrate and placebo groups.^15^ This approval sets a promising precedent for the development of future PPARα agonists, highlighting their potential for safety, tolerability, and efficacy in the treatment of related conditions. However, pemafibrate has not been approved in the USA by the FDA.

Recently, we reported a potent and highly isotype-selective nonfibrate PPARα agonistic chemotype based on the 4-benzyloxy-benzylamino scaffold. Given the premise described previously, we became interested in the potential of this series for CIPN indications. The most advanced compound in this series is A190 (Figure S1), which exhibits an EC_50_ < 40 nM in cell-based assays and a >2700-fold selectivity for PPARα over PPAR-δ and PPAR-γ.^16^ A190, however, exhibits poor aqueous solubility (0.028 mg/mL), resulting in reduced oral bioavailability, expected to dampen therapeutic efficacy in CIPN relevant contexts. The lack of solubility was anticipated to lead to a lack of dose proportionality, difficulty in maintaining steady-state plasma concentrations, and undesirable side effects. These limitations underscore the need to develop drug delivery systems that could address the issues of this promising chemotype.

Oral delivery remains the preferred route of drug administration but faces challenges such as poor solubility, low permeability, gastrointestinal instability, and presystemic metabolism.^17^ Most recently, strategies have focused on enhancing solubility or incorporating drugs into nano- or microparticle systems like microemulsions, which offer advantages such as high drug loading, small droplet size, and self-dispersing properties.^18^ Microemulsions are thermodynamically stable disperse systems developed using an optimal concentration of oils, surfactants, and cosurfactants. This system can enhance the oral bioavailability of hydrophobic drugs by stimulating pancreatic and biliary secretions, increasing gastrointestinal transit time, stimulating lymphatic transport, increasing intestinal membrane permeability, and reducing the activity of metabolism and efflux pumps.^19^ Additionally, their small droplet size (<200 nm) increases surface area, further enhancing drug absorption and distribution.^20^

Considering these premises and the potential of microemulsions, we hypothesized that a new oil-in-water (o/w) microemulsion of A190 could improve solubility and intestinal membrane permeability of A190, increase oral bioavailability, and enhance therapeutic efficacy against CIPN. To test this hypothesis, we first selected the microemulsion components based on drug solubility and excipient miscibility and then rationally optimized the A190 microemulsion formulation. Subsequently, extensive studies were conducted to analyze the physicochemical characteristics of the formulation, assess in vitro and ex vivo permeability, evaluate pharmacokinetic profiles in healthy rats, and determine the in vivo efficacy of A190 microemulsion in CIPN in mice. We also further evaluated the efficacy on another important complete Freund’s adjuvant (CFA)-induced chronic inflammatory pain mouse model.^21^

## 2 Materials and Methods

### 2.1 Materials

Paclitaxel (purity >98%) was purchased from Athenex, NDC 70860-200-50, Richmond, USA. Paclitaxel was dissolved in a 1:1:18 mixture of 200 proof ethanol, kolliphor, distilled water, Tween 80, polyethylene glycol 400 (PEG 400), propylene glycol, CFA, dimethyl sulfoxide (DMSO), and (2-hydroxypropyl)-β-cyclodextrin (HP-β-CD), was purchased from Sigma-Aldrich, MO, USA. Fenofibrate d6 (internal standard, IS, purity >95%) was purchased from Toronto Research Chemicals, New York, ON, Canada. MK886 was purchased from Tocris (1311), Bristol, United Kingdom, and dissolved in a 1:1:18 mixture of 200 proof ethanol, kolliphor, and distilled water (Sigma-Aldrich). Caprylocaproyl macrogol-8-glycerides (Labrasol), diethylene glycol monoethyl ether (Transcutol HP), and propylene glycol monocaprylate (Capryol 90) were provided as a gift by Gattefossé, Saint-Priest, France. Caco-2 cells (human colon cancer) and HepG2 cells (human hepatocellular carcinoma) obtained from American Type Culture Collection (ATCC, Manassas, VA, USA).

### 2.2 Animals

Sprague-Dawley rats (males, 250–300 g) were purchased from Charles River Laboratories (Wilmington, MA) for in vivo pharmacokinetic study. Pain model experiments were conducted using adult (10–15 weeks) male and female C57BL/6J mice from Jackson Laboratory (Bar Harbor ME, USA). Initially, they were maintained in a temperature- and humidity-controlled vivarium space (21 ± 3 °C, 55 ± 10%) on a 12 h light/dark cycle (lights on at 7:00 AM) with free access to food (Teklad LM-485 mouse sterilized diet, Harlan Laboratories Inc., Indianapolis IN, USA) and water until needed. Then mice were retrieved from the vivarium and housed (4–5 mice per cage) for the duration of the study in a temperature- and humidity-controlled out-of-vivarium space on the same light/dark cycle. They were given ad libitum food and water. All experiments were performed during the light cycle. This study was approved by the Institutional Animal Care and Use Committee of Virginia Commonwealth University and carried out in accordance with the National Institutes of Health’s Guide for the Care and Use of Laboratory Animals.

### 2.3 Preparation and Characterization of A190-Loaded Microemulsions

#### 2.3.1 Solubility-Based Selection of Microemulsion Components

An essential criterion for selecting microemulsion components is the solubility of poorly water-soluble drugs. An excess amount of A190 was added to 1 mL of various aqueous phases (deionized water, phosphate-buffered saline (PBS), PBS with 0.2% Tween 80, and PBS with 0.2% sodium lauryl sulfate (SLS)), oils (oleic acid, Capryol 90, and Maisine oil), and surfactants or cosurfactants (Labrafil M 1944 CS, PEG 400, Tween 80, Transcutol HP, Labrasol, propylene glycol, Cremophor) in stoppered glass vials. Each sample was vortexed and kept in an isothermal shaker maintained at 25 ± 1.0 °C to reach equilibrium for 48 h. The resulting mixture was centrifuged at 4000*g* (Eppendorf 5424 R Refrigerated Centrifuge) for 15 min, and the supernatant was collected and diluted with acetonitrile followed by filtration with 0.22 μm (PTFE, hydrophobic, Thermo Scientific) membrane filters. The concentration of A190 in the filtrates was quantified by high-performance liquid chromatography with ultraviolet (HPLC-UV) (Shimadzu Prominence LC system). Chromatographic separation was achieved using an Agilent Pursuit XRs 5 C18 column (250 × 4.6 mm) with a UV-detector system. The mobile phase consisted of acetonitrile and water, both containing 0.1% trifluoroacetic acid in a ratio of 70:30 v/v, with a flow rate of 1 mL/min at 25 °C. The injection volume was 10 μL. The detection was performed at a 227 nm wavelength. After selection of oils and surfactants/cosurfactants with higher A190 solubility, miscibility studies were performed as previously described.^22^ In brief, selected excipients were mixed with each other in equal volumes (1 mL), vortexed for 10 min, and left to equilibrate for around 30 min. The resulting mixture was visually inspected for transparency, turbidity, and phase separation.

#### 2.3.2 Optimization Using Box–Behnken Experimental Design

Several process and formulation parameters integral to the microemulsion formulation might influence the final physicochemical characteristics, drug loading, and stability of the microemulsion. Therefore, a Box–Behnken design (BBD) with 3-factor and 3-level (3^3^) was conducted to screen the most relevant process parameters and determine the minimum number of experiments needed to optimize A190 microemulsion. The total of 15 experimental runs was generated and evaluated using JMP Pro 14 software (SAS Institute, Cary, NC, USA), as shown in Table 2. Based on preliminary studies, the three key parameters influencing the physicochemical properties of A190 microemulsion, namely, percentages of oil (*X*_1_), surfactant/cosurfactant ratio (*X*_2_), and sonication time (*X*_3_), were selected as independent variables. All of the independent variables were adjusted at three different levels: −1 (lower level), 0 (medium level), and +1 (higher level). Additionally, mean microemulsion droplet size (*Y*_1_), polydispersity index (PDI; *Y*_2_), zeta potential (*Y*_3_), and drug loading (*Y*_4_) were taken as the responses for the design experiment. In addition, physical stability of the prepared microemulsions, such as phase separation and transparency/turbidity was evaluated. To evaluate the reproducibility of the design, 3-center points were included, whereas the impact of unexplained variability was minimized by conducting all experiments in a random order. A statistical model for each response was chosen based on the highest order polynomial, where additional terms are significant, and the model is not aliased. The optimized A190 microemulsion was prepared according to the predictor profile for maximum desirability within the design space. The model optimization was based on achieving A190 microemulsion with minimum droplet size and PDI with maximum drug loading percentage and zeta potential values between −10 and +10 mV.

A190 microemulsion with different concentrations of oils (7.5 to 22.5%), surfactant/cosurfactant mixtures of 40% with 3:1, 1:1, and 1:3 v/v ratios, and deionized water (37.5% to 52.5%) were prepared using a high-energy ultrasonication method. In brief, A190 was dissolved in oleic acid (oil) under sonication (Branson CPX2800H, USA) for 30 min, and a fixed weight ratio of Tween 80/PEG 400 (surfactant/cosurfactant mixture) was added. After vigorous mixing, deionized water was slowly added with continuous stirring at 1500*g* for 15 min. The emulsions formed were sonicated by ultrasonic Liquid Processor VCX 500 (Sonics & Materials, Inc., CT, USA) at an amplitude of 40%. Finally, each A190 microemulsion was subjected to physicochemical characterization.

#### 2.3.3 Characterization of the A190 Microemulsions

The mean droplet size, PDI, and zeta potential of the A190 microemulsion were measured by using a dynamic laser light scattering analyzer (Malvern Zetasizer Nano ZS90; Malvern Instruments, Malvern, UK). All A190 microemulsion formulations were diluted with 10 mM NaCl solution at a ratio of 1:100 v/v and sonicated for 1 min to minimize multiple scattering effects. Each measurement carried out at 25 °C was estimated by averaging three runs and presented as mean ± standard deviation (SD). The drug content in the A190 microemulsion was determined by diluting each formulation with acetonitrile, filtering using a 0.2 μm membrane filter, and quantifying using the HPLC system with a UV detector, as mentioned earlier in Section 2.3.1. The droplet morphology and droplet size of the A190 microemulsion were confirmed using high-resolution cryo-transmission electron microscopy (cryo-TEM). In brief, the A190 microemulsion was diluted 100 times in deionized water, and grids were prepared using a FEI Vitrobot Mark IV. 3.5 μL of sample was applied to the grid before blotting and plunging into liquid ethane. Blot force 5 with 10 s of blotting time was applied. They were imaged using a Thermo Scientific Glacios at 200 kV with an XFEG electron source and a Falcon4 direct electron detector.

#### 2.3.4 In Vitro Dissolution Study

A dissolution study was performed using a USP I Apparatus (Basket) (Sotax AT Xtend, Switzerland) with 100 mL of medium containing 0.1 N HCl solution (pH 1.2) or PBS pH 6.8 at 100 rpm and a temperature of 37 ± 0.2 °C. In each test, A190 dispersion or A190 microemulsion (3.75 mg in 1.5 mL) was filled in a hydroxypropyl methylcellulose capsule (size 00) from Qualicaps. Capsules were placed inside the basket and subjected to a dissolution test, and 1 mL of samples was withdrawn at 10, 20, 30, 45, 60, 90, 120, 180, 240, and 300 min. In the meantime, the same volume of fresh medium was added to keep a constant volume. The dissolution study of each sample was performed in triplicate. After filtration, the amount of A190 in the collected dissolution samples was quantified by the HPLC method, as described in Section 2.3.1.

#### 2.3.5 Stability of A190 Microemulsions

The optimum formulation selected from the BBD was subjected to the thermodynamic stability tests, including the centrifugation test, heating–cooling cycle test, and freeze–thaw test.^23^ To understand the effect of centrifugal force on phase separation of two immiscible phases (water and oil), A190 microemulsion was centrifuged down at 1000*g* for 30 min (Eppendorf 5424 R Refrigerated Centrifuge). For the heating–cooling cycle test, A190 microemulsion was examined at room temperature for 48 h, after being subjected to 6 cycles of heating (45 °C) and cooling (4 °C). Whereas, for freeze–thaw cycles, A190 microemulsion was examined at room temperature for 48 h, after being subjected to 3 cycles of freezing (−21 °C) and thawing (25 °C). In each test, A190 microemulsion was studied for phase separation, creaming, or cracking. To further access the stability of the microemulsion, the dispersibility study was performed using USP 2 dissolution apparatus. In brief, 1 mL of A190 microemulsion was dispersed in 100 mL of PBS pH 6.8 at 37 °C and observed for phase separation and drug precipitation as previously described.^24^

To assess the storage stability, an optimum A190 microemulsion was placed in glass scintillation vials and kept at room temperature (25 ± 5 °C) for 3 months. At a specified time period, the microemulsion formulation was evaluated in terms of droplet size, PDI, zeta potential, and the percentage of drug content remaining. In addition, the A190 microemulsion was visually inspected for signs of instability, including phase separation, turbidity, transparency, precipitation, and color change. Each experiment was performed in triplicate, and the results were presented as the mean ± SD.

### 2.4 In Vitro and Ex Vivo Permeability of A190 Microemulsion

To predict the passive intestinal permeability of a drug, the parallel artificial membrane permeability assay (PAMPA) technique is used, which utilizes a phospholipid-coated lipophilic membrane that mimics the properties of the intestinal wall.^25^ The in vitro intestinal membrane permeability of free A190 (dispersed in PBS pH 6.8), A190 dispersion (dispersed in a mixture of 10% PEG 400 + 5% DMSO + 85% of 10% HP-β CD), and A190 microemulsion was evaluated using a PAMPA (BD BioSciences, San Jose, CA, USA), as described previously.^22,26^ Briefly, donor samples were prepared by the dilution of free A190, A190 dispersion, and A190 microemulsion with PBS at pH 6.8 at a concentration of 200 μg/mL (based on A190). After loading 0.3 mL of PBS at pH 6.8 to each well of the acceptor plate, both the acceptor and donor plates were sandwiched, while ensuring that the membrane of the donor plate was in proper contact with the media in the acceptor plates. Next, 0.2 mL of the diluted samples was loaded in each well of the donor plate. Following incubation of the entire plate for 5 h at room temperature, the plate assembly was separated, and samples were collected from both the acceptor and donor plates. The concentration of A190 that permeated through the phospholipid membrane was measured by HPLC as described earlier. The effective permeability (*P*_e_) of each drug was then calculated using the following formula
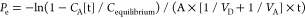
where *P*_e_ is the permeability (cm/s), *A* is the effective filter area (0.228 cm^2^), *V*_D_ is the volume of the donor well (0.2 mL), *V*_A_ is the volume of the receptor well (0.3 mL), *t* is the total time of incubation in seconds, *C*_A_(*t*) denotes the concentration of drug in the receptor well at time *t*, and *C*_equilibrium_ represents (*C*_D_[*t*] × *V*_D_ + *C*_A_[*t*] × *V*_A_)/(*V*_D_ + *V*_A_), where *C*_D_(*t*) denotes the concentration of drug in the donor well at time t.

Ex vivo intestinal permeation study of the optimized A190 microemulsion, A190 dispersion, and free A190 was carried out using freshly excised rat intestinal membrane via the noneverted rat sac model.^27^ In brief, SD rats housed in standard conditions with free access to food and water were sacrificed, and the duodenal part of the small intestine was excised and cleaned for facial debris and mucus using Ringer’s lactate solution (ICU Medical, Inc.). The clean intestinal segment was cut into pieces of 6 cm length, and one end was tied firmly to form a sac. Optimized A190 microemulsion, A190 dispersion, and free A190 diluted to 1 mg/mL in PBS pH 6.8 were loaded in the sac (0.5 mL), and the second end of the intestinal segment was tied using thread. The sac was placed in a jacketed glass with 25 mL of PBS pH 6.8, maintained at 37 °C with continuous stirring (100 rpm). At predetermined time intervals, 1 mL of release media was collected and replaced with fresh media. The concentrations of A190 permeated through the noneverted intestinal sac were quantified using the HPLC method, as described in Section 2.3.1. The apparent permeability coefficient (*P*_app_) of A190 or A190 microemulsion was calculated using the formula
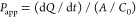
where *P*_app_ is the apparent permeability (cm/s), d*Q*/d*t* is the rate of linear appearance of mass on the receptor side (μmoL/s), *C*_0_ is the initial concentration of A190 inside the sac (μg/mL), and *A* is the surface area of the intestinal sac exposed to the A190 microemulsion or dispersion. Surface area was calculated by treating the intestinal segment as a cylinder and using the formula: *A* = 2π × radium × length.

### 2.5 Cell Viability Studies

To evaluate the cytotoxicity of free A190 (in 0.2% DMSO in DMEM), vehicle microemulsion, and A190 microemulsion against Caco-2 cells and HepG2 cells, MTT assay using 3-(4,5-dimethylthiazol-2-yl)-2,5-diphenyltetrazolium bromide was performed. Caco-2 and HepG2 cells were cultured in a 25 cm^2^ tissue culture flask with DMEM supplemented with 10% FBS and penicillin (100 U/mL), streptomycin (100 μg/mL) at 37 °C in a humidified incubator with 5% CO_2_. Once the cells reached confluence, they were trypsinized, and 100 μL of cell suspension at a density of 10 × 10^4^ cells/well was seeded in 96-well tissue culture plates, followed by incubation at 37 °C. After 48 h, the growth medium was replaced with freshly prepared dilutions of free A190, vehicle microemulsion, and A190 microemulsion diluted in DMEM (0.1, 1, 2.5, 5, 7.5, 10, 25, 50, and 100 μM based on A190). Untreated cells (DMEM) and cells treated with 0.2% DMSO in DMEM served as controls. After 24 and 48 h of incubation, the media from each well were replaced with 100 μL of MTT solution (5 mg/mL in PBS, filtered). The plates were gently shaken and then incubated at 37 °C in a humidified 5% CO_2_ incubator for 3 h. Following the incubation, the supernatant was removed, and 100 μL of DMSO was added to dissolve the formazan crystals. The DMSO was gently pipetted up and down to ensure the complete solubilization of crystals. Absorbance was then measured by using a plate reader at 540 nm. The cell viability percent of each group was calculated using the following equation: (optical density of samples/optical density of control) × 100.

### 2.6 In Vivo Oral Absorption in Rats

To evaluate the improvement in the oral bioavailability of A190 after formulation as a microemulsion, we orally administered A190 microemulsion to rats. Each rat received either the A190 dispersion or the A190 microemulsion at a dose of 20 mg/kg. Subsequently, 150 μL of blood samples was collected via tail vein at predetermined intervals and transferred in sodium EDTA-coated tubes. The blood was immediately centrifuged (2500*g*, 15 min, 4 °C), and the plasma was separated and stored at −80 °C until analysis.

To measure the plasma concentration of A190, a total of 100 μL of each standard solution or defrosted plasma sample were spiked with 100 μL of fenofibrate d6 (5 μg/mL, IS). Next, 400 μL of ice-cold acetonitrile was added to the mixture to precipitate protein, followed by vortex mixing and centrifugation at 9000*g* for 5 min at 4 °C. After centrifugation, the supernatant was transferred to a glass vial and evaporated to dryness using a sample concentrator dry-block (Techne, Cambridge, UK) at 50 °C. Finally, residues were reconstituted with 100 μL of acetonitrile/water (1:1, v/v). The plasma concentration of A190 was determined using a LC mass spectrometer (LC-MS-2020) (Shimadzu Corporation) with a Phenomenex Kinetex C18 column (100 × 2.1 mm, 1.7 μm, PFP 100 Å). The chromatographic separation was performed using isocratic mobile phase (acetonitrile with 0.1% formic acid: water with 0.1% formic acid, 60:40, v/v) at a flow rate of 0.3 mL/min. A 10 μL sample was injected, and A190 was measured in positive ionization mode using an electron spray ionization interface. The following parameters were optimized for A190 analysis: interface temperature, 350 °C; DL temperature, 250 °C; nebulizing gas flow, 1.5 L/min; drying gas flow, 12 L/min.

### 2.7 Chemotherapy-Induced Peripheral Neuropathy Pain Model

CIPN model was established as previously described.^28^ In brief, paclitaxel was administered at a dose of 8 mg/kg intraperitonially every other day; four administrations completed one injection cycle. Control mice received paclitaxel dissolved in a mixture of 1:1:18 v/v/v (ethanol/kolliphor/distilled water) at a dose of 10 mL/kg, i.p., and followed the same injection cycle. Animals began behavioral testing 14 days after the final injection. A190 microemulsion was administered at a dose of 10 and 20 mg/kg by oral gavage and tested at different time points (1, 3, 6, 24, 48, and 72 h) after drug administration. To investigate whether paclitaxel-induced hypersensitivity reduction with oral dosing of A190 microemulsion was mediated through PPARα activation, CIPN mice were pretreated with the selective PPARα antagonist, MK886 (6 mg/kg, i.p., 30 min pretreatment time), followed by A190 microemulsion (20 mg/kg, p.o.), and mice were tested 48 h later.

### 2.8 Induction of Chronic Inflammatory Pain by CFA

We explored the effects of A190 microemulsion in a widely used model of persistent inflammatory pain, which is based on the injection of CFA into the paw. CFA is composed of inactivated and dried *Mycobacterium tuberculosis* and adjuvant and was purchased from Sigma-Aldrich (St. Louis, MO, USA). The CFA pain model is based on hypersensitivity, paw swelling, and nuclear factor-κB-mediated transcription of tumor necrosis factor α involved in the formation of the principal mediators of inflammation.^29^ Mice were injected intraplantarly with 20 μL of CFA (50%, diluted in mineral oil; Sigma-Aldrich). Mechanical sensitivity (see the measurement of the von Frey test) was measured before and 3 days after the CFA injection. A190 microemulsion (20 mg/kg) or vehicle microemulsion was administered by oral gavage on day 4 after CFA injection, and mice were tested for mechanical sensitivity at different time points (1, 3, 6, 24, 48, and 72 h) after drug injection.

#### 2.8.1 Evaluation of Mechanical Sensitivity

Mechanical sensitivity thresholds were determined according to the method of Chaplan et al., and as adapted in Toma et al., 2017.^28,30^ A series of calibrated von Frey filaments (Stoelting, Wood Dale IL, USA) with logarithmically incremental stiffness ranging from 0.07 to 3.6 expressed as diameter sensitivity (ds) log 10 of 10 × force (in milligrams) were applied to the paw with a modified up-down method.^30^ The mechanical threshold was expressed as log 10 of 10 × force (in milligrams), indicating the force of the von Frey hair to which the animal reacted (paw withdrawn, licking, or shaking). All behavioral testing on animals was performed in a blinded manner.

#### 2.8.2 Paw Edema Measurement

Immediately following Von Frey testing, paw edema was measured. The thickness of CFA-treated paws was measured 48 h after drug administration using a digital caliper (Traceable Calipers, Friendswood, TX). Data were recorded to the nearest ±0.01 mm and expressed as change in paw thickness (ΔPD = difference in the ipsilateral paw diameter – contralateral paw thickness).

#### 2.8.3 Locomotor Activity

Mice were placed into individual Omnitech photocell activity cages (28 cm × 16.5 cm) (Columbus OH, USA) 24 h after administration of either vehicle or A190 microemulsion (20 mg/kg, p.o.). Interruptions of the photocell beams (two banks of eight cells each) were then recorded over 60 min. Data are expressed as the number of photocell interruptions.

### 2.9 Histopathology Evaluation

SD rats were administered daily doses of either a vehicle microemulsion or a A190 microemulsion for 3 consecutive days. On day 4, rats were euthanized using CO_2_ euthanasia followed by cervical dislocation. For histological evaluation, various tissues, including the liver, heart, spleen, kidney, duodenum, jejunum, and ileum were immediately fixed in 10% neutral buffered formalin for 24 h before being transferred to 70% ethanol. The tissues were then embedded in paraffin wax and sectioned into 5 μm-thick slices using a microtome. Hematoxylin and eosin staining was performed on the tissue sections. A blinded pathologist evaluated the stained slides for any signs of tissue damage, inflammation, or other histological alterations. Imaging was conducted using Phenochart, a tissue imaging software integrated with the Phenoimager (AKOYA Biosciences).

### 2.10 Statistical Analysis

Behavioral data are expressed as the mean ± SEM (standard error of the mean). Time-course data and other behavioral results were analyzed using one-way or two-way repeated measures of variance (ANOVA), followed by posthoc Tukey tests with the alpha level set at 0.05. The behavioral statistical analysis was performed with GraphPad Prism software, version 9.5 (GraphPad Software, Inc., La Jolla, CA, USA). The probability was considered significant if *P* < 0.05. No significant sex differences were observed in all experiments, so the male and female data were pooled. For all other analysis, one-way or two-way ANOVA followed by Tukey’s multiple-comparison test was used to compare more than two mean values. All data were expressed as the mean ± SD for in vitro analysis and mean ± SEM for in vivo analysis. In all analyses, *p* < 0.05 was considered statistically significant.

## 3 Results

### 3.1 Preparation and Characterization of A190 Microemulsions

**Figure 1:**
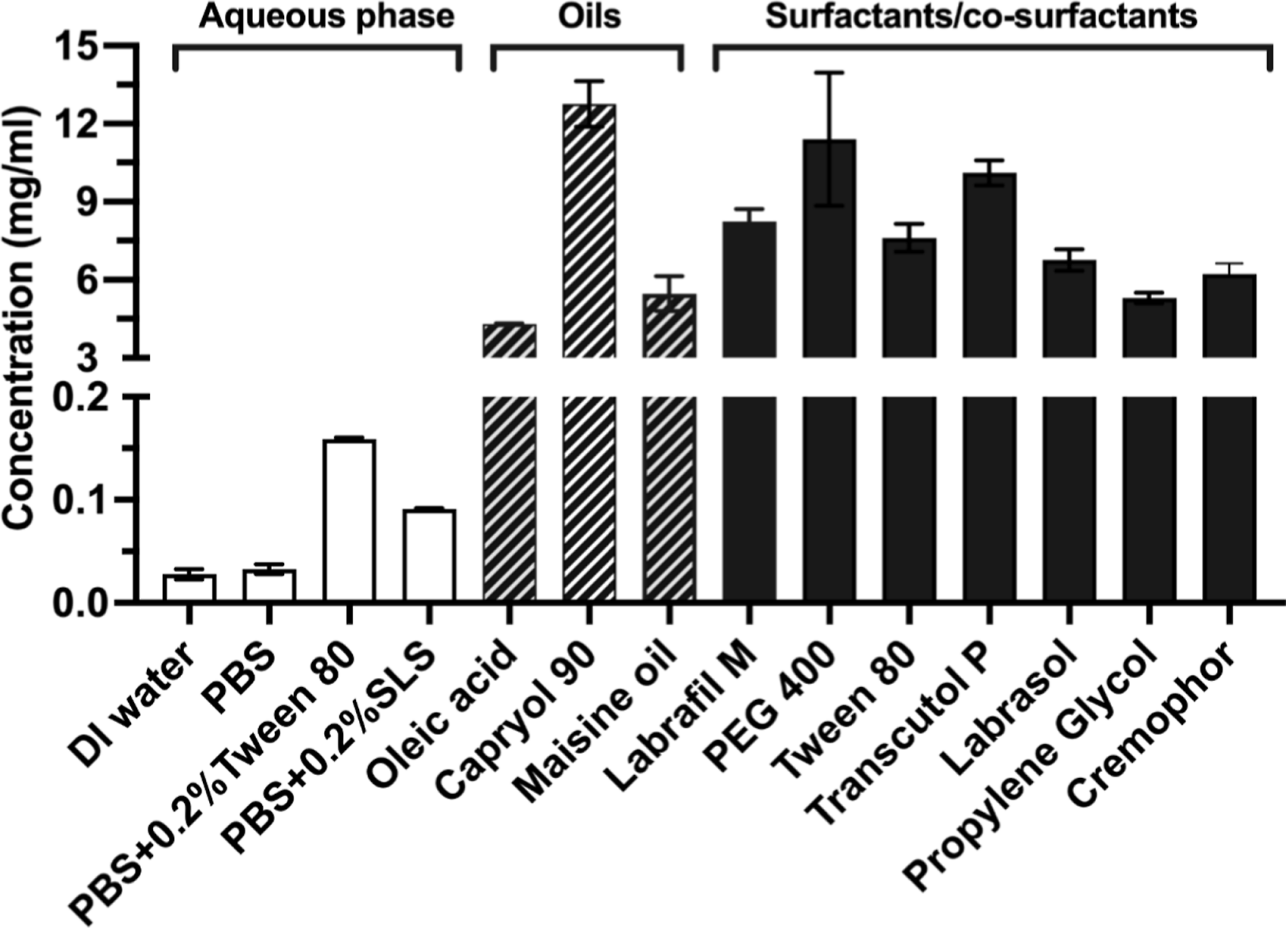
Solubility of A190 in various oils, surfactants, and cosurfactants. Each value represents the mean ± SD (*n* = 4 for each group).

Solubility studies of A190 were conducted to identify appropriate oils, surfactant, and cosurfactant for the formulation of a stable A190 microemulsion, as shown in Figure 1. The solubility of A190 in Capryol 90 (12.7 ± 0.88 mg/mL) was higher compared to oleic acid (4.3 ± 0.03 mg/mL), and Maisine oil (5.5 ± 0.67 mg/mL). However, oleic acid was selected as the oil phase, based on the solubility along with good miscibility with the chosen surfactants and cosurfactants. A190 was more soluble in PEG 400 (13.7 ± 2.56 mg/mL), Transcutol HP (10.5 ± 0.48 mg/mL), and Tween 80 (8.07 ± 0.53 mg/mL) compared to Labrasol (6.39 ± 0.41 mg/mL), propylene glycol (5.47 ± 0.20 mg/mL), and Cremophor (6.62 ± 0.40 mg/mL). The surfactant and cosurfactant that demonstrated higher solubility with appropriate miscibility were subsequently selected as components for the design of the experiment using BBD. Besides, the solubilities of A190 in aqueous medium, such as deionized water, PBS, PBS with 0.2% Tween 80, and PBS with 0.2% SLS, were 0.028 ± 0.01, 0.033 ± 0.005, 0.159 ± 0.001, and 0.091 ± 0.001 mg/mL, respectively.

**Figure 2:**
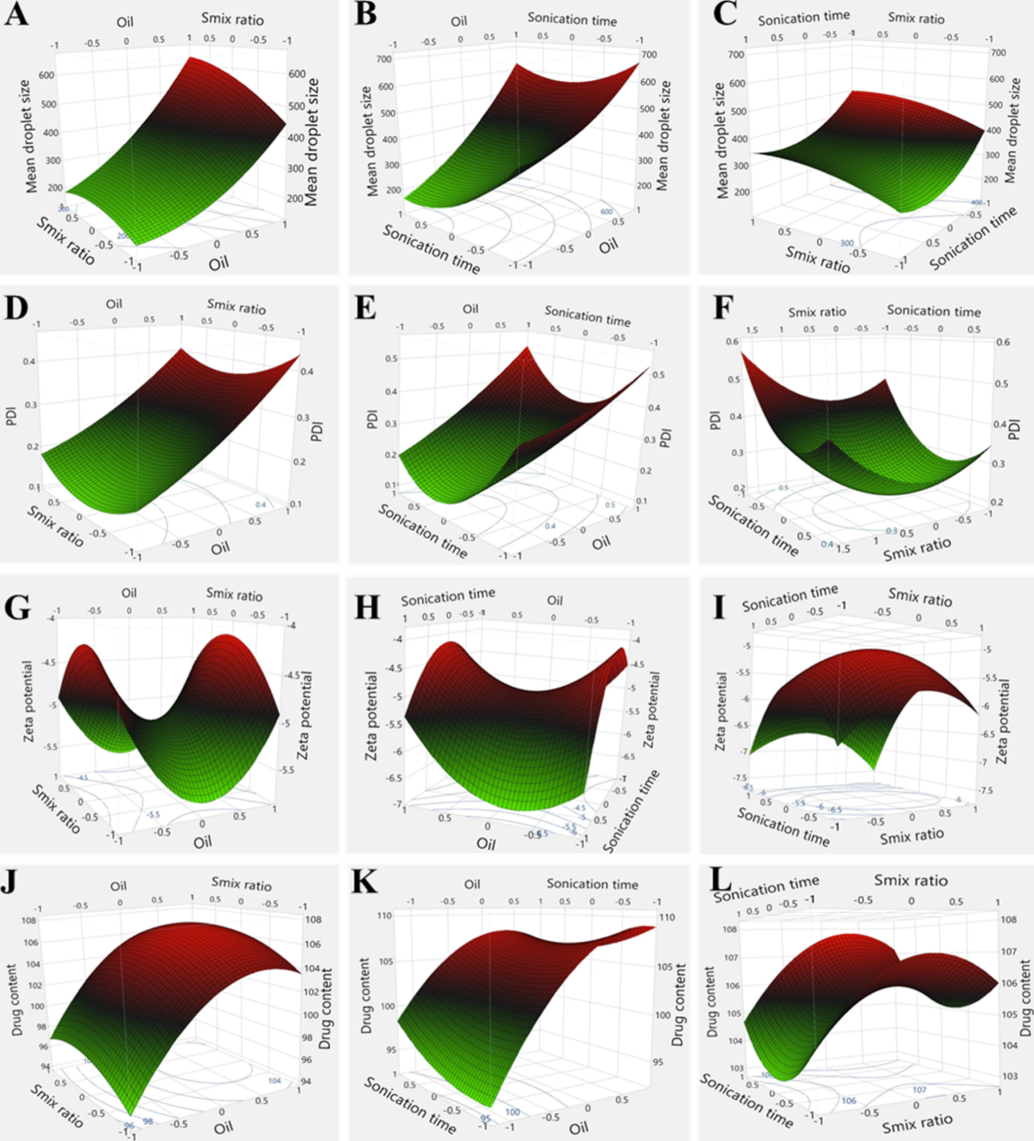
3D response surface plots, including contour plots as generated by BBD, representing the effect of independent variables on dependent variables. (A,D,G,J) Effect of oil and *S*_mix_ ratio on mean droplet size, PDI, zeta potential, and drug content, respectively; (B,E,H,K) effect of oil and sonication time on mean droplet size, PDI, zeta potential, and drug content, respectively; and (C,F,I,L) effect of *S*_mix_ ratio and sonication time on mean droplet size, PDI, zeta potential, and drug content, respectively.

Following the preliminary study to select oil, surfactant/cosurfactant, a 3-factor, 3-level response surface-based BBD design was run to screen and select the critical process and formulation-related factors that affect the mean droplet size, PDI, zeta potential, and drug content. The details of the experimental run with composition, process parameters, and the corresponding responses obtained are shown in Table 1. The outcomes for each dependent variable were used to determine the model’s best fit, using the corresponding *F*- and *p*-values. In addition, the lack of fit and regression coefficient was calculated, thereby providing insights into the reliability and precision of the selected model. The mean droplet size, PDI, zeta potential, and drug content of the A190 microemulsion ranged from 148 to 686 nm, 0.135 to 0.593, −7.7 to −3.7 mV, and 95.5 to 108.1%, respectively, across the different trials, depending on the composition and levels of each factor specified by the design (Table 2). The ANOVA results for the BBD showed that the model for mean droplet size (*Y*_1_), PDI (*Y*_2_), and drug content (*Y*_4_) were significant with the *F*-values of 7.97, 3.36, and 4.68 with *p*-values of <0.05 each, and *R*^2^ of 0.950, 0.984, and 0.957, respectively (Table S1). In addition, *Y*_1_, *Y*_2_, and *Y*_4_ were significantly affected by the percent of oil (*X*_1_) as indicated with the *p*-values <0.01, <0.05, and <0.01, respectively. In addition, the mean droplet size was affected by sonication time (*Y*_3_) but not by the *S*_mix_ ratio (*Y*_2_). No significant effect of *S*_mix_ ratio and sonication was observed on PDI. Whereas the model for zeta potential (*Y*_3_) was nonsignificant with *F*-value of 1.45, *p*-value of 0.36, and *R*^2^ of 0.957 (Table S1). 3D response surface plots were used to study the influence of the main and interactive effects of independent variables on mean droplet size, PDI, zeta potential, and drug content, as shown in Figure 2A–C,D–F,G–I,J,K, respectively. When two independent variables were varied over a certain range, one variable was kept constant. The shape of the 3D response plots in Figure 2A,D,J revealed that the increase in oil concentration in the microemulsion formulation positively affected on the mean droplet size, PDI, and drug content. Besides, the nonsignificant lack of fit values for each of these models also proved that the selected model was significant (Table S1).

**Table 1:** List of Dependent and Independent Variables in BBD for A190 Microemulsion

independent variables	levels		
	low (−1)	medium (0)	high (+1)
factor 1 (*X*_1_): oil (%)	7.5	15	22.5
factor 2 (*X*_2_): surfactant/cosurfactant ratio (*S*_mix_ ratio) (v/v)	3:1	1:1	1:3
factor 3 (*X*_3_): sonication time (min)	0	1.5	3
dependent variables	constraints		
response 1 (*Y*_1_): mean droplet size	minimum		
response 2 (*Y*_2_): PDI	minimum		
response 3 (*Y*_3_): zeta potential	in range		
response 4 (*Y*_4_): drug content	maximum		

**Table 2:** Design of Experiment Using BBD to Study the Combined Effect of Independent Variables on Mean Droplet Size, PDI, Zeta Potential, and Drug Content

run	independent variables			responses			
	*X*_1_	*X*_2_	*X*_3_	*Y*_1_	*Y*_2_	*Y*_3_	*Y*_4_
	oil (%v/v)	*S*_mix_ ratio	sonication time (min)	mean droplet size (nm)	PDI	zeta potential (mV)	drug content (%)
#1	7.5	1:3	1.5	173.3 ± 0.8	0.169 ± 0.007	–3.7 ± 0.3	95.7 ± 3.0
#2	7.5	1:1	0	395.0 ± 3.5	0.431 ± 0.093	–5.0 ± 0.1	96.2 ± 2.0
#3	7.5	1:1	3	148.8 ± 1.8	0.135 ± 0.053	–6.6 ± 0.2	102.1 ± 4.4
#4	7.5	3:1	1.5	221.1 ± 1.1	0.186 ± 0.015	–5.1 ± 0.2	95.5 ± 0.7
#5	15	1:3	0	420.8 ± 22.4	0.416 ± 0.255	–7.2 ± 0.4	104.5 ± 1.3
#6	15	1:3	3	272.1 ± 2.7	0.257 ± 0.039	–7.7 ± 0.7	102.6 ± 5.7
#7	15	1:1	1.5	216.1 ± 3.0	0.168 ± 0.016	–4.6 ± 0.7	103.0 ± 1.4
#8	15	1:1	1.5	392.1 ± 11.6	0.237 ± 0.108	–5.8 ± 0.4	107.3 ± 1.4
#9	15	1:1	1.5	388.5 ± 8.4	0.471 ± 0.053	–4.9 ± 0.1	107.1 ± 1.3
#10	15	3:1	0	480.6 ± 24.2	0.403 ± 0.209	–5.6 ± 0.4	108.1 ± 0.2
#11	15	3:1	3	326.3 ± 3.8	0.138 ± 0.063	–6.9 ± 0.4	107.2 ± 1.8
#12	22.5	1:3	1.5	405.4 ± 12.7	0.430 ± 0.014	–4.8 ± 0.2	104.8 ± 5.7
#13	22.5	1:1	0	686.7 ± 9.7	0.593 ± 0.004	–6.0 ± 0.2	107.7 ± 3.7
#14	22.5	1:1	3	647.6 ± 39.0	0.251 ± 0.079	–4.9 ± 0.2	107.2 ± 0.4
#15	22.5	3:1	1.5	627.9 ± 48.0	0.413 ± 0.275	–5.6 ± 0.5	104.7 ± 4.0

**Figure 3:**
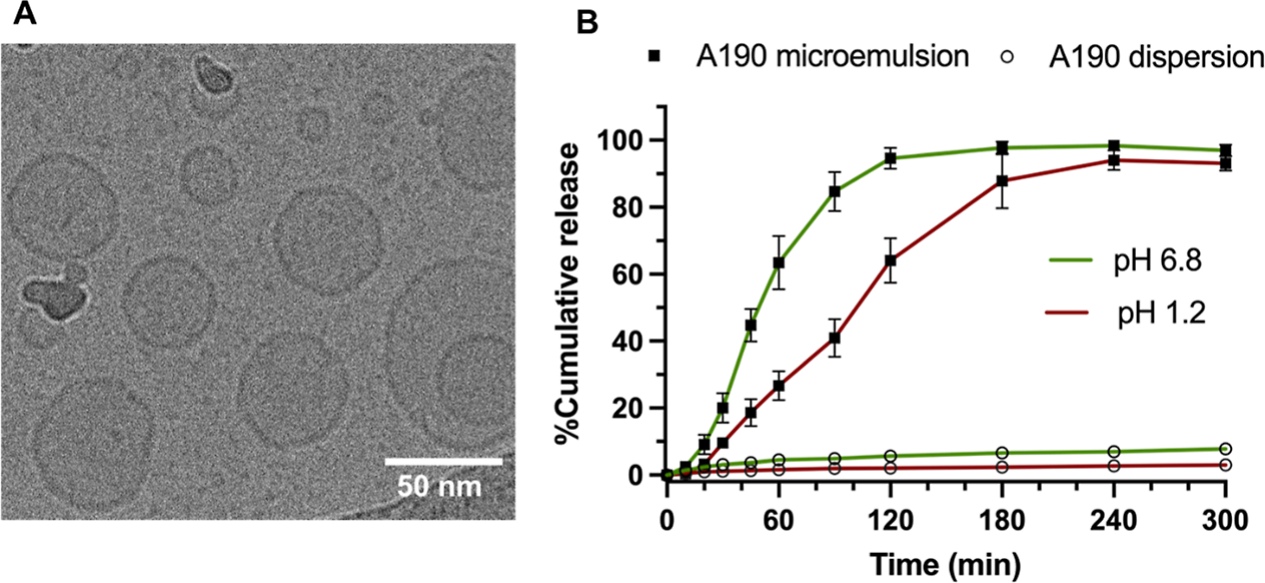
(A) Cryo-TEM image of A190 microemulsion. (B) In vitro cumulative percentage release profiles of A190 dispersion (in 10% PEG 400 + 5% DMSO + 85% of 10% HP-β CD mixture) or A190 microemulsion in pH 1.2 or pH 6.8 media. Each value represents the mean ± SD (*n* = 3 for each group).

The A190 microemulsion was selected from the prediction profiler of the BBD. Using this profiler, we set the values of independent variables so that the optimum microemulsion formulation would have a minimum mean droplet size and PDI, zeta potential near neutral (−10 to +10 mV), and drug content close to 100%. The optimum formulation consisted of 7.5% of oil, 40% of *S*_mix_ (1:1 v/v), and 52.5% of aqueous phase. The placebo microemulsion had a mean droplet size, PDI, and zeta potential of 119.8 ± 1.64 nm, 0.131 ± 0.007 nm, and −5.92 ± 0.87 mV, respectively. The mean droplet size, PDI, and zeta potential of the A190-loaded optimum microemulsion remained similar even after the incorporation of A190, which indicates that A190 was well distributed in the oil phase (Table S2). The A190 content in the optimized microemulsion as measured using HPLC showed the drug content of >95%. Figure 3A shows the cryo-TEM image of A190 microemulsion, which confirmed the formation of spherical nanosized droplets with a narrow size distribution, consistent with the results obtained with a laser diffraction particle analyzer.

To verify the self-emulsification property of the A190 microemulsion, we performed an in vitro dissolution study of the A190 dispersion and microemulsion in 0.1 N HCl solution (pH 1.2) or phosphate buffer (pH 6.8) (Figure 3B). In a dissolution medium at pH 1.2, approximately 68% of A190 was released from the microemulsion within 120 min, while the dispersion formulation showed less than 3% dissolution. From the observation (Figure 3B), it is clear that the drug release was faster at pH 6.8 compared to that at pH 1.2, highlighting the impact of pH on the release profile of the drug. At pH 6.8, only 5.5% of A190 was dissolved from the dispersion, compared to >90% released from the microemulsion at 120 min. These finding indicates that the microemulsion formulation released the majority of the drug within 120 min, significantly enhancing the dissolution rate of the highly lipophilic A190. This is because the A190 microemulsion facilitated successful self-emulsification in the dispersion medium, preventing phase separation or drug precipitation.

Microemulsions are thermodynamically stable systems; therefore, A190 microemulsion was studied for freeze–thaw cycle test, centrifugation test, and heating–cooling cycle test. No phase separation, precipitation, creaming, and loss of drug content was observed for A190 microemulsion, which confirmed the formation of thermodynamically stable microemulsion. Moreover, A190 microemulsion passed the dispersibility test with no phase separation and precipitation observed in PBS media. Long-term stability of microemulsion is a critical parameter to be considered during formulation optimization. Therefore, we performed the long-term storage stability of the optimized A190 microemulsion at room temperature (25 ± 5 °C). Upon 3 months of storage, the drug content of A190 microemulsion was maintained at >95%, and no significant change in the mean droplet size, PDI, zeta potential, and drug content was recorded (Figure S2). In addition, the optimized formulation was physically stable with no signs of phase separation and precipitation.

### 3.2 In Vitro and Ex Vivo Permeability of A190 Microemulsion

To understand the passive diffusion of free A190, A190 dispersion, and A190 microemulsion through an artificial intestinal membrane, we performed in vitro permeability using an artificial phospholipid membrane. The *P*_e_ of the A190 dispersion was higher by 1.5-fold compared to free A190. Moreover, the incorporation of A190 into microemulsion formulation significantly increased the effective permeability of A190 by 8.6- and 13.1-fold compared to A190 dispersion and free A190, respectively (Table 3). Moreover, the apparent permeability for the optimized A190 microemulsion formulation was 12.2 × 10^–6^ cm/s, which was significantly greater by 2-folds compared to that of A190 dispersion (6.0 × 10^–6^ cm/s) (Table 3). However, free A190 did not permeate through the intestinal membrane (below the limit of quantification, 150 ng/mL).

**Table 3:** Effective Permeability and Apparent Permeability of A190, A190 Dispersion, and A190 Microemulsion^a^

sample	effective permeability across artificial membrane (*P*_e_, ×10^–6^, cm/s)	apparent permeability across intestine (*P*_app_, ×10^–6^, cm/s)
A190 in PBS	0.09 ± 0.02	
A190 dispersion	0.13 ± 0.04	6.01 ± 1.19
A190 microemulsion	1.16 ± 0.20^***, ###^	12.20 ± 2.89^*^

a Notes: values are mean ± SD (*n* = 9–12). ***P < 0.001, compared to A190 in dispersion; ^###^*P* < 0.001, compared to A190 in PBS pH 6.8; *P < 0.05, compared to A190 in dispersion.

### 3.3 In Vitro Cytotoxicity

**Figure 4:**
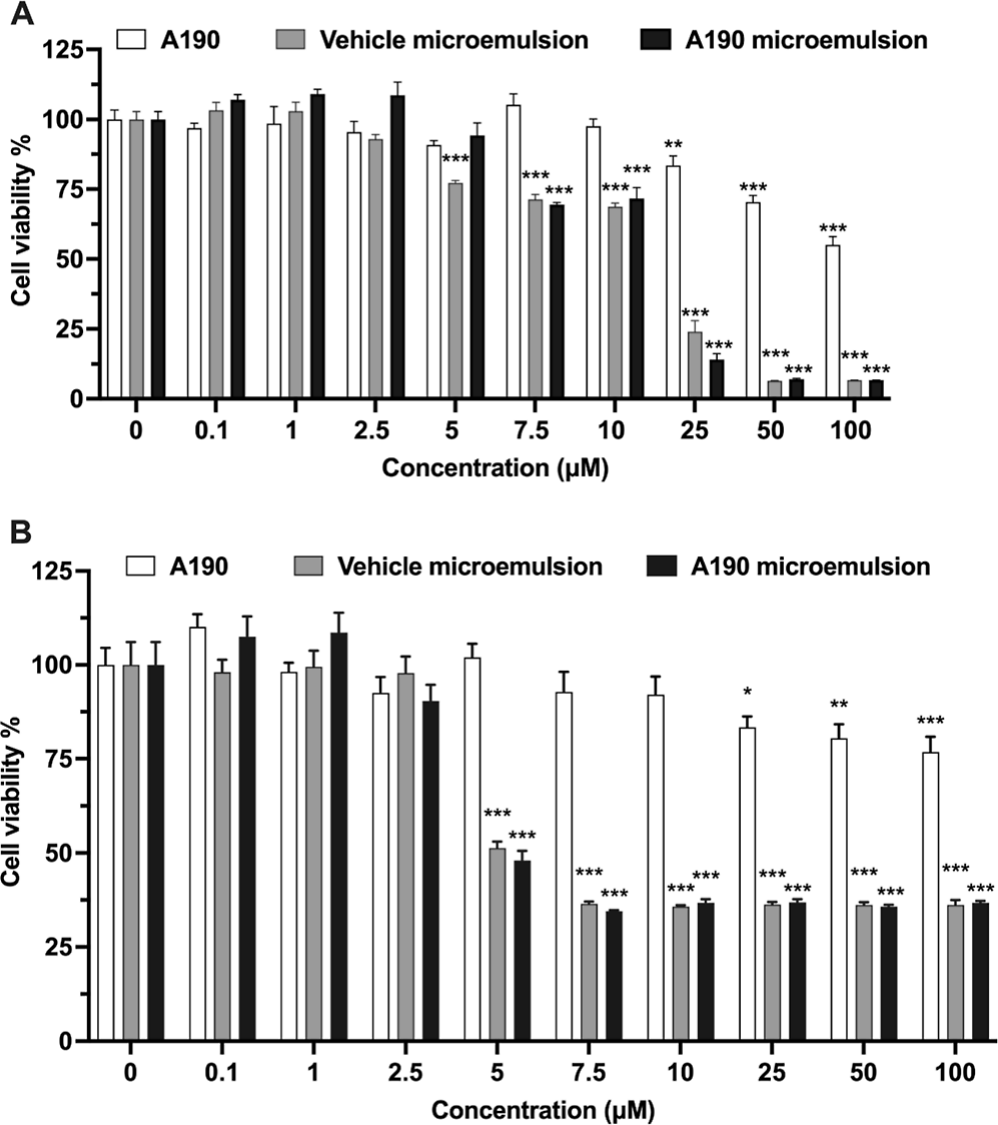
In vitro cytotoxic effects of A190 in 0.2% DMSO, vehicle microemulsion, and A190 microemulsion on (A) HepG2 cells and (B) Caco-2 cells after incubation for 24 h. Values are mean ± SD (*n* = 5). *p < 0.05, **p < 0.01, ***p < 0.001 compared to respective DMEM or 0.2% DMSO controls.

To assess the potential cytotoxic effects of free A190, vehicle microemulsion, and A190 microemulsion, an MTT assay using Caco-2 and HepG2 cell lines was performed (Figure 4). Caco-2 cells, derived from human colon carcinoma, differentiate upon ∼3 weeks of culture to form a polarized monolayer with microvilli on the apical side of the cell membrane and form tight junctions between adjacent cells. These characteristics make them an excellent model to mimic the small intestinal epithelium and evaluate the permeability of drugs across the intestinal barrier.^31^ Meanwhile, HepG2 cells, a human liver-derived cell line, are used to assess the potential hepatotoxicity and metabolic processing of drugs, given the liver’s pivotal role in drug metabolism.^32^ No significant cytotoxicity was observed for free A190 in 0.2% DMSO, even at the highest concentration of <50 μM in both cell lines at 24 (Figure 4A,B) and 48 h (Figure S3). The biocompatibility of the prepared microemulsion was evaluated by assessing the cytotoxicity of the vehicle alone (no drug). The vehicle microemulsion had no significant influence on the cell viability (>80%) of Caco-2 and HepG2 cells within the concentration range of 0–5 μM, indicating less toxicity and biocompatibility of the materials used, as shown in earlier studies.^33^ In each cell line, the A190 microemulsion maintained cell viability of more than 80% at a concentration below 5 μM (Figure 4). However, concentration-dependent toxicity was observed at a higher concentration.

### 3.4 Pharmacokinetics Study

**Figure 5:**
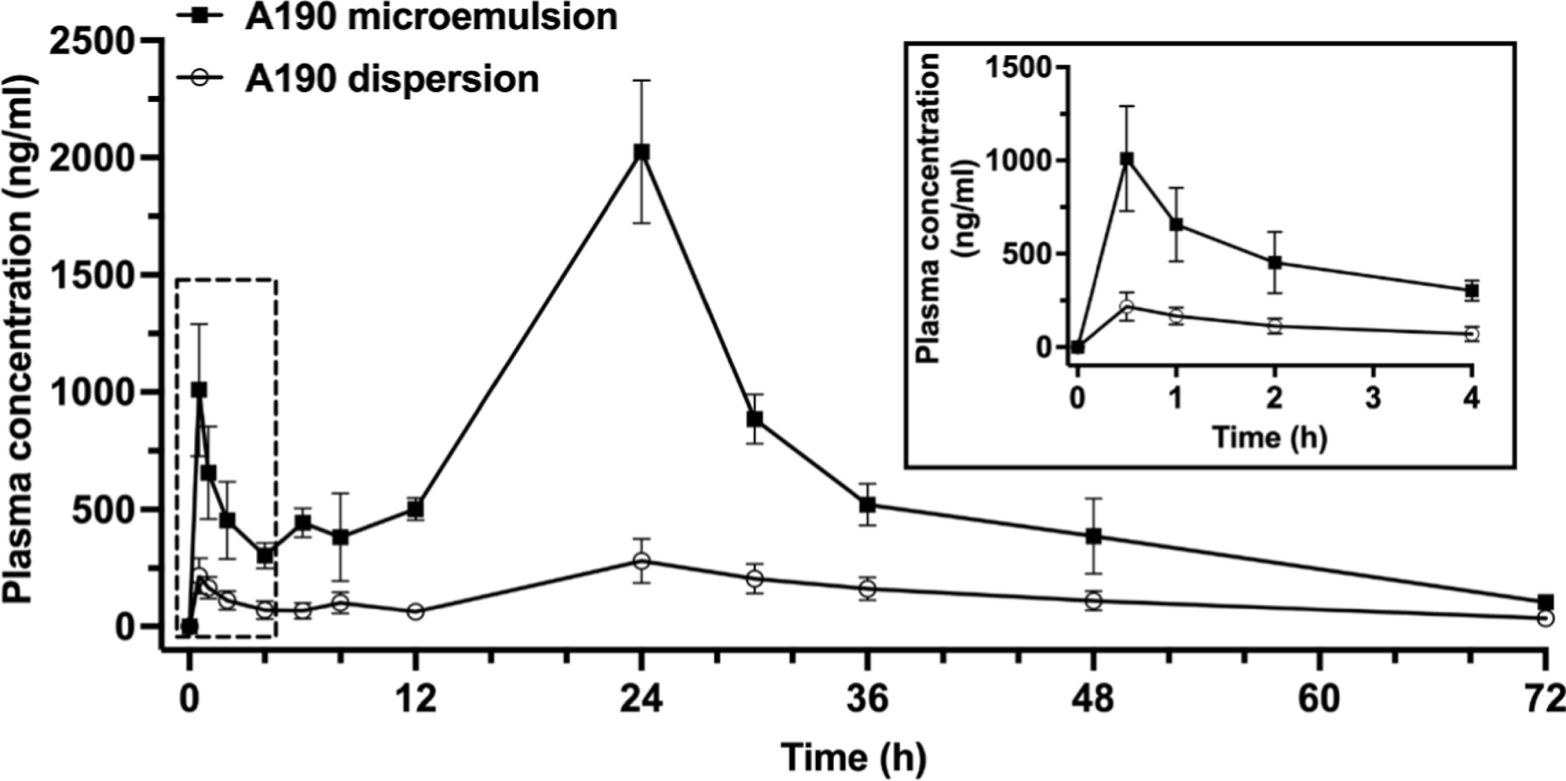
Mean plasma concentration–time profiles of A190 after oral administration of A190 dispersion (20 mg/kg) and A190 microemulsion (20 mg/kg) to rats. Each value represents the mean ± SEM (*n* = 4–5 for each group). Insert graph represents enlarged segment from 0 to 4 h.

An in vivo absorption study was conducted to assess whether the improved solubility and enhanced permeability of A190 could enhance its oral bioavailability. Figure 5 shows the plasma concentration profiles of A190 after oral administration of the A190 dispersion and A190 microemulsion to rats. The pharmacokinetic parameters are summarized in Table 4. The AUC of orally administered A190 microemulsion was 9980.9 ± 2621.0 ng/mL h, which was significantly higher by 4.7-fold compared with that of A190 dispersion (47,434.9 ± 4824.9 ng/mL*h). The *C*_max_ was also increased by 5.06-fold in the A190 microemulsion compared to that of orally administered A190 dispersion (2024.8 ± 527.9 vs 400.8 ± 105.7 ng/mL). Both formulations demonstrated a delayed *T*_max_ of ≥24 h. In addition, the oral bioavailability of A190 from the microemulsion was 4.9-fold higher compared to orally administered A190 dispersion. No difference in the *t*_1/2_ was observed between the A190 microemulsion and the A190 dispersion. These findings indicated that the oral bioavailability of the hydrophobic drug could be substantially enhanced by using the o/w microemulsion formulation strategy.

**Table 4:** Plasma Pharmacokinetic Parameters of A190, Following Oral Administration of A190 Dispersion and A190 Microemulsion to Rats (Mean ± SD, *n* = 4–5)^a^

formulation	A190 dispersion	A190 microemulsion
dose (mg/kg)	20	20
*t*_max_ (h)	27.6 ± 4.8	24.00 ± 0.0
*t*_1/2_ (h)	14.5 ± 4.01	15.6 ± 6.1
*C*_max_ (ng/mL)	400.8 ± 105.7	2024.8 ± 527.9^*^
AUC_0→*t*_ (ng/mL*h)	9140.2 ± 2268.4	44,769.3 ± 4693.3^*^
AUC_inf_ (ng/mL*h)	9980.9 ± 2621.0	47,434.9 ± 4824.9^*^
relative bioavailability (%)	1	4.9 ± 2.3^*^

a AUC_inf_, area under the curve from time zero to infinity; AUC_0→*t*_, area under the curve from time zero to the last sampling time point; *C*_max_, maximum observed concentration; *t*_1/2_, half-life. Statistical analysis: Student’s *t*-test. *P < 0.05 compared to A190 dispersion.

### 3.5 Effects of A190 Microemulsion in the CIPN Model

**Figure 6:**
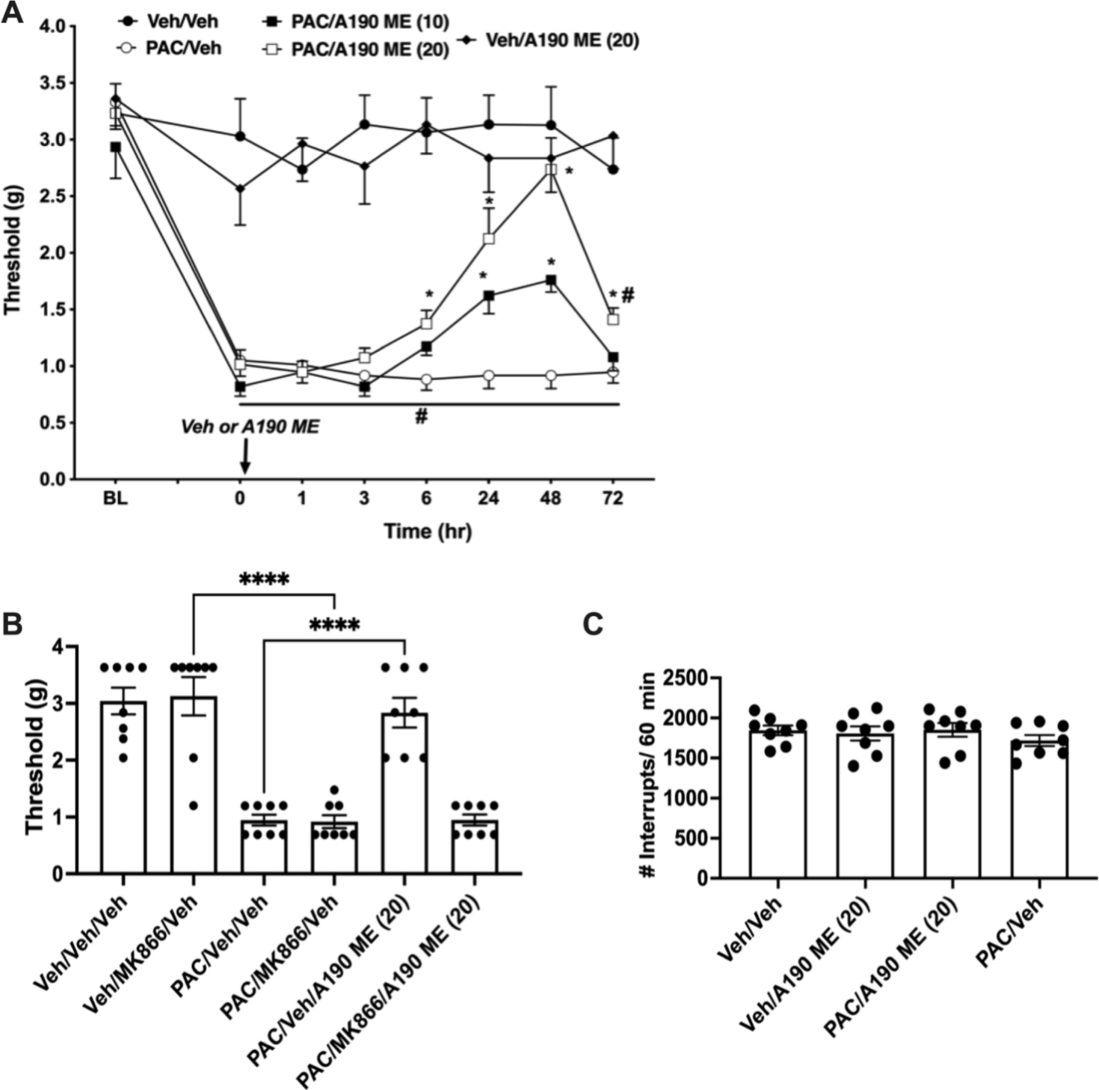
Effects of A190 microemulsion (A190 ME) in the CIPN model. (A) Effects of systemic A190 microemulsion in the CIPN pain model. Animals began behavioral testing 14 days, following the final injection of paclitaxel. Antiallodynic effects after oral administration of A190 microemulsion (20 mg/kg) were measured. Mechanical paw withdrawal thresholds were determined at 1, 3, 6, 24, 48, and 72 h after the drug administration. (B) A190 microemulsion did not significantly impact paw withdraw thresholds with mice pretreated with MK886 compared to their vehicle group (*P* < 0.0001) in the CIPN model. (C) At the highest dose tested, A190 microemulsion (20 mg/kg) did not significantly alter locomotor activity compared to vehicle-treated animals (*P* = 0.5947). Data are expressed as the mean ± SEM (*n* = 8/group; 50% male and 50% female). #*p* < 0.05 vs veh/veh group, *p < 0.05 vs PAC/veh group. Veh = vehicle; PAC = paclitaxel; and ME = microemulsion.

A190 microemulsion was tested at doses of 10 and 20 mg/kg in the CIPN model. As reported in Figure 6, A190 microemulsion reduced CIPN-induced mechanical hypersensitivity as shown in a 2-way repeated ANOVA analysis with *F* (treatment) (4, 35) = 71.46; *P* < 0.0001), *F* (time (4.844, 169.5) = 29.11; *P* < 0.0001), and (*F* treatment × time (28, 245) = 4.343; *P* < 0.0001), where *F* (treatment) denotes the dose of A190 microemulsion administered. Posthoc analysis (Tukey) revealed that the dose of 20 mg/kg partially reversed CIPN-induced mechanical hypersensitivity 6 to 72 h after administration, while the lowest dose 10 mg/kg of A190 microemulsion partially reversed CIPN-induced mechanical hypersensitivity from 24 to 48 h after administration. The effect of the A190 microemulsion at 10 mg/kg dissipated 48 h after administration (Figure 6A). In vehicle-treated mice 20 mg/kg (no PAC), A190 did not alter von Frey responses. In addition, an ordinary one-way ANOVA analysis showed that A190 microemulsion did not significantly impact paw withdraw thresholds (F (5,42) = 28.75; *P* < 0.0001) (Figure 6B), with mice pretreated with a known PPARα inhibitor (MK886), compared to their vehicle group. Finally, at the highest doses tested, A190 (20 mg/kg) did not significantly alter locomotor activity compared to vehicle-treated animals (F (3, 28) = 0.6416; *P* = 0.5947) (Figure 6C).

### 3.6 Activity of A190 in the CFA-Induced Inflammatory Pain Model

**Figure 7:**
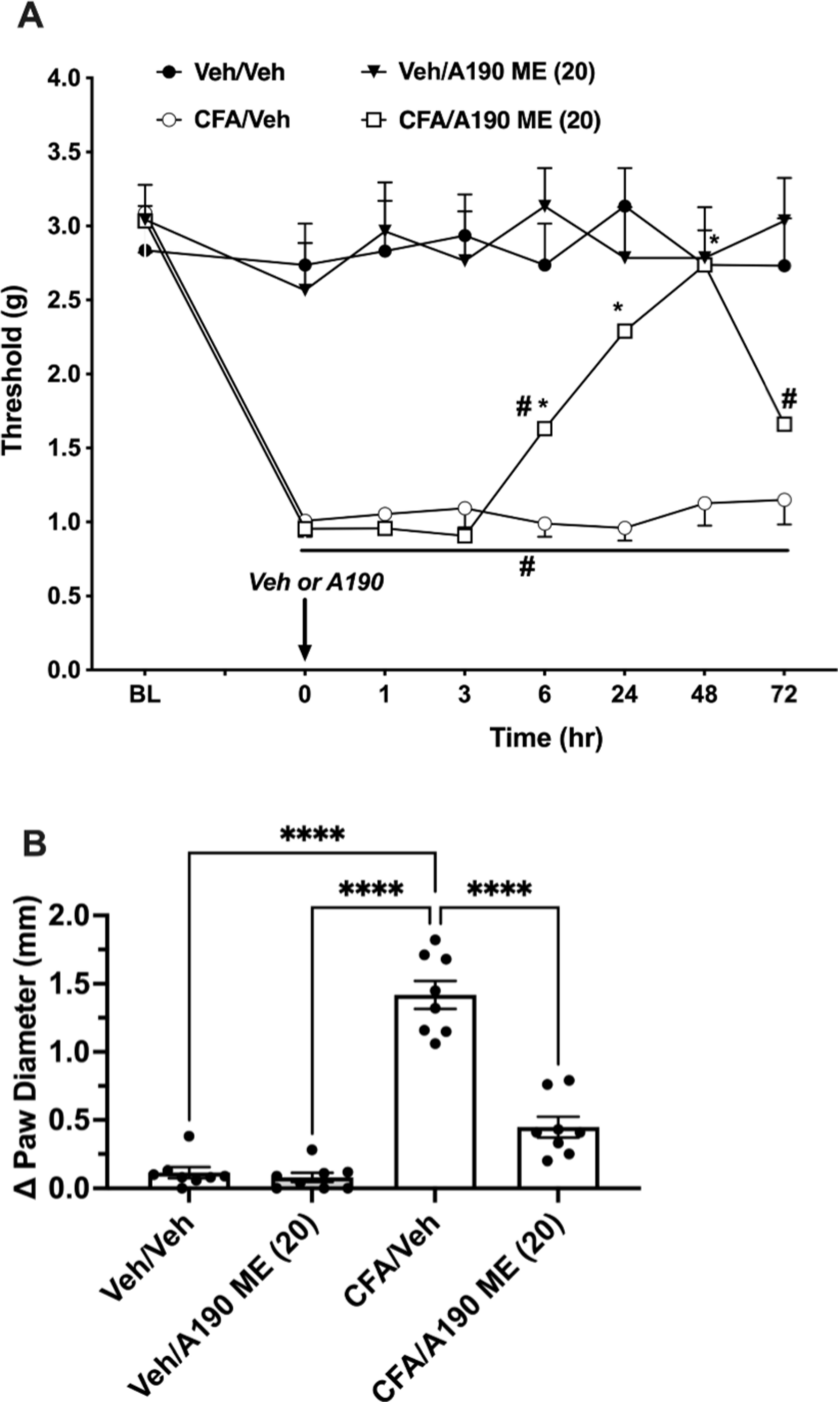
Effects of A190 microemulsion (A190 ME) in the chronic inflammatory CFA model. (A) Effects of systemic A190 microemulsion in the CFA model. Antiallodynic effects after oral administration of A190 (20 mg/kg). Mechanical paw withdrawal thresholds were determined 3 days after intraplantar injection of CFA (100%) at 1, 3, 6, 24, 48, and 72 h after the drug administration (*n* = 8/group; 50% male and 50% female). A190 fully reversed the mechanical hypersensitivity in a time-related manner. (B) Systemic A190 microemulsion (20 mg/kg) reduces paw edema at 48 h after administration (*P* < 0.0001). ΔPaw diameter = ipsilateral paw diameter – contralateral paw diameter. Data are expressed as the mean ± SEM #*p* < 0.001 vs veh/veh group and *#*p* < 0.05 vs CFA/veh group. Veh = vehicle; ME = microemulsion.

A190 microemulsion was tested at 20 mg/kg in the CFA model. As reported in Figure 7, A190 microemulsion reduced CFA-induced mechanical hypersensitivity as shown in a 2-way repeated ANOVA analysis with *F* (treatment) (3, 28) = 62.63; (*P* < 0.0001), *F* time (5, 145) = 9.736; (*P* < 0.0001) and (*F* treatment × time (21, 196) = 4.286; *P* < 0.0001), where *F* (treatment) denotes the dose of A190 microemulsion administered. Posthoc analysis (Tukey) revealed that the dose of 20 mg/kg partially reversed CFA mechanical hypersensitivity 6 h and fully at 48 h after injection. The effect of the A190 microemulsion dissipated 72 h after administration (Figure 7A). In vehicle-treated mice 20 mg/kg, A190 did not alter von Frey responses. In addition, A190 microemulsion significantly reduced paw edema (*F* (3, 28) = 81.06; *P* < 0.0001, Figure 7B), with mice treated with 20 mg/kg dose differing from the vehicle group.

### 3.7 Histological Evaluation

**Figure 8:**
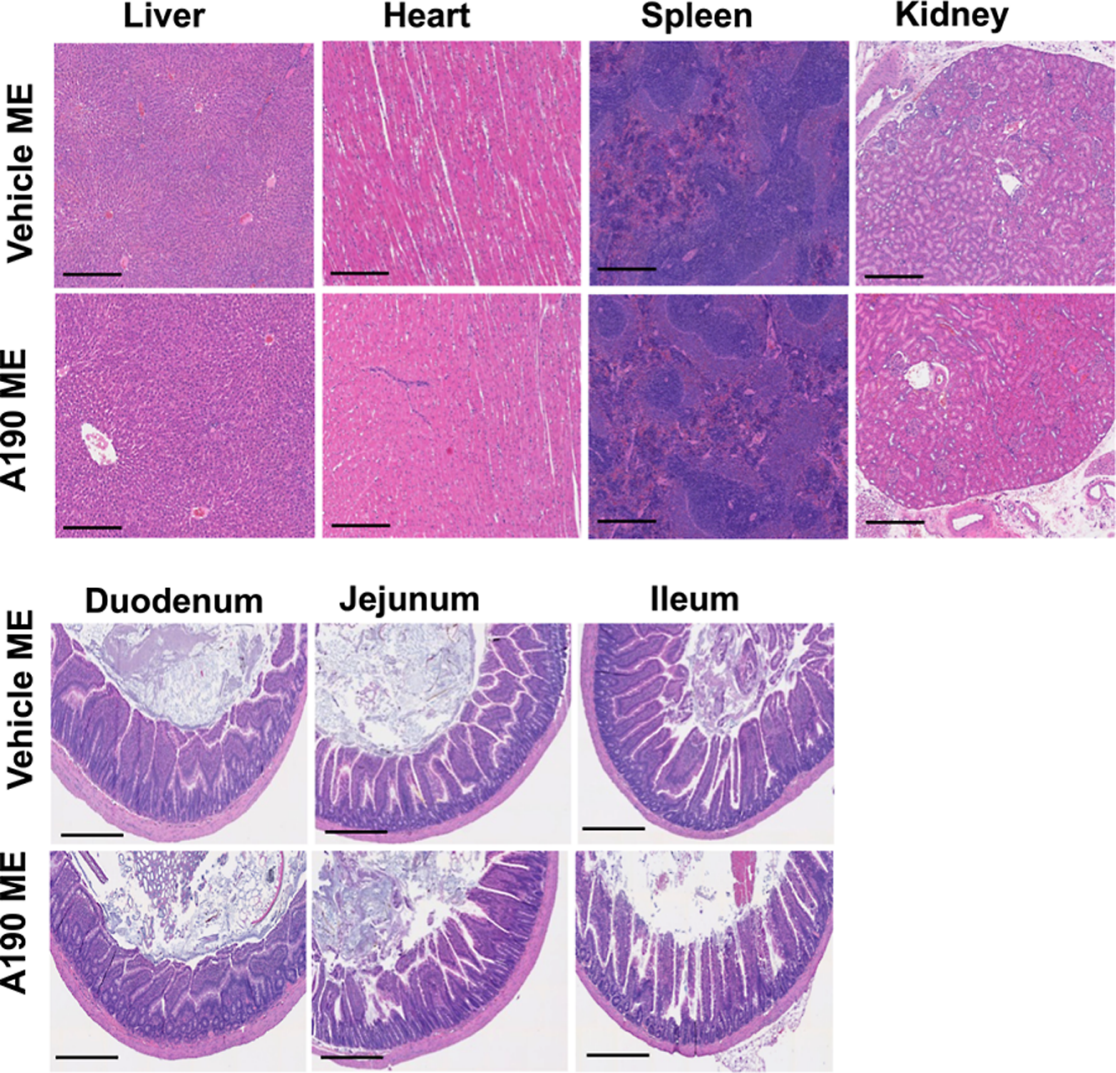
Representative histological images of tissues (liver, heart, spleen, kidney, duodenum, jejunum, and ileum) after oral administration of vehicle ME and A190 ME (20 mg/kg once daily for 3 days). Scale bar: 200 μm.

The safety and tolerability of oral formulations are critical for ensuring minimal systemic toxicity, maintaining intestinal integrity, and achieving the effective bioavailability of the drugs. The histopathological evaluations of various tissues such as the liver, heart, spleen, kidney, duodenum, jejunum, and ileum, showed no evidence of toxic effects after 3 days administration of vehicle microemulsion and A190 microemulsion (Figure 8). The hepatic lobules appeared uninjured with no signs of inflammation or pathological alterations. The myocardium showed no evidence of fibrosis, and the cardiac myocytes displayed a typical cytoarchitecture without any signs of hypertrophy. The spleen tissues exhibited a normal architecture, with clearly defined red and white pulp. Renal tissues displayed intact glomerular and tubular structures with no evidence of sclerosis or acute necrosis. In addition, the duodenum, jejunum, and ileum showed no clinically significant histological or morphological alterations at the administered dose.

## 4 Discussion

Oral drug delivery can be significantly affected by low aqueous solubility and limited intestinal permeability, reducing bioavailability and therapeutic efficacy. To address these limitations, novel formulations such as nanoemulsions, microemulsions, lipid- or polymeric systems, and prodrugs strategies, are emerging as promising solutions.^34,35^ The microemulsion-based system offers biocompatibility, high drug loading, sustained release, and enhanced efficacy by maintaining drug concentrations at the target site while reducing toxicity.^36^ Using microemulsion technology, Novartis developed Neoral, the first oral peptide formulation of cyclosporine (a highly lipophilic cyclic polypeptide), which showed superior pharmacokinetic properties and reduced variability in absorption, resulting in reduced pharmacokinetic variability.^37^ To date, there is no published report on the effectiveness of an orally administered PPARα agonist-loaded microemulsion in the treatment of peripheral neuropathic pain.

In the present study, we developed an A190 loaded microemulsion with the drug dissolved in the oil phase, which acts as a drug reservoir and prolongs drug release. All excipients used in the microemulsion formulation were classified as “generally recognized as safe”. The aqueous solubility of A190 was <30 μg/mL at 25 °C (Figure 1). Thus, the formulation of A190 microemulsion required appropriate selection of oil based on the drug’s solubility and its miscibility with the surfactant/cosurfactant, while surfactant and cosurfactants are selected based on solubilizing capacity and the ability to stabilize the microemulsion.^38^ The solubility of A190 in oleic acid was lower than that of Capryol 90 (Figure 1). However, oleic acid was selected as the oil phase due to its amphiphilic nature, which contributes to the reduction of the interfacial tension, facilitating the formation of stable microemulsion.^39^ Tween 80 was selected as a pH- and ionic strength-resistant surfactant, while PEG 400, as a cosurfactant, can minimize drug precipitation and enhance the diffusivity of the formulation.^36,40^

We optimized the A190 microemulsion using BBD, which allowed us to identify an optimized microemulsion formulation from a minimal number of trials by statistically varying process parameters.^41^ Preliminary studies revealed that the concentration of oil and the ratio of surfactant/cosurfactant can significantly impact the droplet size and PDI of the microemulsion formulation.^22^ To further investigate the effects of these independent variables along with sonication time on the droplet size, PDI, zeta potential, and drug content of the A190 microemulsion, we employed a three-factor, three-level (3^3^) BBD with 15 experimental runs (Table 1). As shown in Figure 2A,D, an increase in oil concentration resulted in an increase in droplet size and PDI, respectively. In addition, according to results of statistical analysis, oil concentration significantly affected the mean droplet size (*p* < 0.01; *F* = 48.70) and PDI (*p* < 0.05; *F* = 14.9) (Table S1). The increase in mean droplet size with increased oil concentration indicates that the interfacial tension is not sufficiently reduced due to limited adsorption of the surfactant molecules on the oil surface.^42^ Additionally, insufficient coverage of oil surface by surfactant molecules leads to oil droplet convergence, thereby increasing coalescence, phase separation, and Ostwald ripening.^43^ This is also a contributing factor to the increased PDI in a microemulsion, indicating a broader size distribution.^44^ Further analysis revealed that altering the ratio of surfactant to cosurfactant initially increased the mean droplet size of the microemulsion, but as the cosurfactant concentration increased, it partially compensated for reduced surfactant levels, stabilizing the droplets and slightly reducing the droplet size and PDI.^45^ These findings emphasize the critical role of balancing formulation components to achieve a stable microemulsion system.

In Figure 2B, an increase in the sonication time decreased the mean droplet size and PDI, which suggests the effect of shear disruptive forces of ultrasound.^46^ Initially, the larger droplets are disrupted, allowing them to coalesce until their surfaces are adequately covered by surfactant molecules for stabilization, with no remaining molecules to form micelles.^47^ All the tested microemulsions exhibited a consistent negative zeta potential (−3.7 to −7.7 mV), which were unaffected by independent variables, aligning with the previous findings (Figure 2G–I). The negative charge was because the surfactant system in the microemulsion formulation remained unchanged and the density of the charged species on the droplet surface stayed constant.^48^ As shown in Figure 2J, increasing the oil concentration enhanced the drug loading capacity by improving drug solubilization, facilitating greater drug partitioning into the oil phase, and expanding the oil droplet surface area, which accommodates more drug molecules.^49^ In this study, no substantial impact of the *S*_mix_ ratio and sonication time on drug content was observed (Figure 2K,L).

The prediction profiler indicated that the optimal formulation and process parameters for A190 microemulsion were: 7.5% of oleic acid, a Tween 80/PEG 400 ratio of 1:1 v/v, and a sonication time of 1.5 min. A190 microemulsions formulated using a high energy ultrasonication, a cost-effective method with superior control over formulation variables compared to micro fluidization and high-pressure homogenization.^50^ The optimized A190 microemulsion showed a monomodal distribution with a low PDI (<0.25) and a mean droplet size of <200 nm with a negative surface charge. Still, the larger average droplet size of A190 microemulsion may result from the bulky side chains of oleic acid and Tween 80, while its low surface charge indicates stabilization predominantly via steric hindrance from the nonionic PEO blocks of Tween 80 rather than electrostatic interactions.^51^ TEM analysis to investigate the microscopic morphology of the A190 microemulsion revealed spherical structures (Figure 3A). These spherical structures might improve cellular uptake, thereby improving the drug delivery efficiency.^52^ The improved aqueous solubility of A190 in microemulsion is crucial for optimal oral bioavailability, as shown by rapid drug release within 120 min in both pH 1.2 and pH 6.8 media. This fast release is attributed to a smaller droplet size, spontaneous emulsification in aqueous media, and gentle agitation mimicking GI motility. Similar findings have been documented in previous studies, which indicated that the formulations with smaller particle sizes exhibit faster drug release rates.^53^

The thermodynamic stability of the optimum A190 microemulsion was confirmed as it remained physically stable and translucent without signs of creaming, cracking, precipitation, or phase separation. This is attributed to the nonionic surfactant/cosurfactant, which lowered the interfacial tension between oil and aqueous phases.^48^ The centrifugation test, heating–cooling cycle, and freeze–thaw cycles had no significant impact on the droplet size or PDI of the microemulsions. During three months of storage at room temperatures, the A190 microemulsion demonstrated good physical stability, with no significant changes observed in the mean droplet size, PDI, and zeta potential (Figure S2). This aligns with previous findings that microemulsions with smaller mean droplet sizes and resistance to flocculation, coalescence, Ostwald ripening, or gravitational separation exhibit physical stability.^54^

In this study, we utilized PAMPA, a noneverted rat sac model, and pharmacokinetic analysis in rats as in vitro, ex vivo, and in vivo methods, respectively, to investigate whether the permeability of a hydrophobic drug-like A190 could be improved using o/w microemulsion as a drug delivery system. Free A190 in PBS (pH 6.8) showed lower permeability compared to that of A190 dispersion, which is due to slightly increased solubilization and improved lipid membrane fluidity caused by PEG 400 and HP-β CD. Moreover, the artificial intestinal permeability and ex vivo study showed a significant increase in the permeability of the A190 microemulsion compared to that of free A190 and A190 dispersion. The improved intestinal permeability can be explained in several ways: (1) A190 microemulsion system enhanced solubilization compared to 10% PEG 400 + 5% DMSO + 85% of 10% HP-β CD mixture and PBS pH 6.8; (2) improved absorption due to its nanoscale size; (3) increased droplets surface area, which enhanced contact with the biological membrane; and (4) absorption-enhancing effects of nonionic surfactants like Tween 80 and PEG 400, which cause reversible disruption of tight junctions and alter intestinal membrane fluidity.^22^

In this study, we investigated the cytotoxic effects of free A190, vehicle microemulsion, and A190 microemulsion on Caco-2 and HepG2 cell lines across concentration ranges from 0.1 to 100 μM. Free A190 exhibited low toxicity (with cell viability remaining above 80% at concentrations of 50 μM or lower) when compared to the vehicle and A190 microemulsion (Figure 4). This lower cytotoxicity, which could be attributed to the safety of the drug molecule or the formation of drug aggregates in the cell culture medium due to A190s high hydrophobicity, which may prevent its adsorption onto the cell lines. Moreover, both Tween 80 and PEG 400 are known to exhibit low cytotoxicity, maintaining high cell viability at lower concentrations.^55,56^

The o/w microemulsion showed promises as an oral delivery vehicle for hydrophobic drug-like A190, as it significantly enhanced the oral bioavailability.^39^ The higher values of *C*_max_ and AUC_last_ when administering the A190 microemulsion compared to the aqueous dispersion indicated the substantial enhancement in the intestinal absorption of the A190 microemulsion, resulting in a 5-fold increase in AUC_last_ without altering its elimination kinetics. This is attributed to the improved solubility of A190 in the microemulsion (>150-fold) and the opening of tight junctions by surfactant/cosurfactant, facilitating paracellular transport, compared to A190 dispersion, which precipitated in the artificial membrane or intestinal membrane.^57^ In the apical side of the small intestine, oil/surfactants stimulate the secretion of pancreatic lipase, bile salts, and bile lipids, which adhere to the surface of microemulsion, forming a more stable emulsion with smaller droplet size that is further hydrolyzed into free fatty acids and monoacylglycerols. These lipid digestion products allow higher solubilization of A190 and may further adhere to gut membranes that passively diffuse across the intestinal epithelial layer, thereby extending GI residence time.^22,58^ The elimination half-lives of A190 dispersion and A190 microemulsion were 14.5 and 15.6 h, respectively, with corresponding *T*_max_ of 27.6 and 24 h. The multiple peaking in pharmacokinetic profiles can speculated to biliary secretion and enhanced enterohepatic recycling efficiency, which reduces clearance and delays the time to reach maximum concentration. The secondary absorption peak likely reflects micelle dissociation, involvement of bile acid, and active reabsorption. To define their contribution, further in vivo experiments using bile duct-ligated animal models in both fed and fasted states need to be performed. Besides, surfactant-stabilized microemulsions, owing to their nanoscale size, may diffuse across the intestinal membrane or be absorbed via clathrin- or caveola-mediated endocytosis and micropinocytosis.^36,39^ As a result, the oral bioavailability of A190 was significantly improved when incorporated into the o/w microemulsion compared to the A190 dispersion, with the relative bioavailability of the A190 microemulsion increasing by 4.9-fold compared to the A190 dispersion.

Chemotherapy drug-like paclitaxel cause peripheral neurotoxicity, limiting their high or cumulative doses. The toxicity is exacerbated when coadministered with other neurotoxic antineoplastic drugs, such as cisplatin, potentially inducing dose-limiting peripheral neuropathy and compromising treatment efficacy.^59^ It has been reported that PPARα plays an important role in the regulation of neuropathic pain.^12^ Our group showed an increase in the expression of PPARα in dorsal root ganglion at day 7 in a CIPN model. However, the early increase in PPARα mRNA expression does not seem adequate to overcome the potential paclitaxel toxicity and alleviate neuropathic pain.^12^ Therefore, to effectively manage inflammation, sustaining elevated PPARα levels over time may be crucial, suggesting that PPARα agonists could represent a promising therapeutic strategy.

In our previous studies, we observed that fenofibrate partially reversed paclitaxel-induced mechanical hypersensitivity and fully reversed cold hypersensitivity, but only when administered intraperitoneally.^12^ This might be due to the limited intestinal absorption and oral bioavailability of fenofibrate. When fenofibric acid, the active metabolite of fenofibrate, was administered, it similarly alleviated hypersensitivity when given either intraperitoneally or orally, although its low aqueous solubility required the use of a vehicle such as sesame oil. Additionally, choline-fenofibrate, a water-soluble salt form, proved to be more effective in reversing both types of hypersensitivity via oral administration without signs of tolerance in animals. Based on these results, the ability of fibrates to reverse paclitaxel-induced mechanical and cold hypersensitivity appears to be PPARα-dependent, as the use of a PPARα antagonist completely blocks the effects of these fibrates.

In this study, we tested the effect of a new PPARα agonist, A190 with enhanced potency and selectivity compared to those of fenofibric acid. A190 microemulsion at 20 mg/kg given orally fully reversed paclitaxel-induced mechanical hypersensitivity, while fenofibric acid given orally at 90 mg/kg partially blocked it.^12^ The results showed a full reduction in mechanical hypersensitivity induced by CIPN and CFA with a single oral administration of the A190 microemulsion. Furthermore, the onset of mechanical hypersensitivity observed between 6 and 72 h after paclitaxel injection aligns with the pharmacokinetic data, which shows that paclitaxel reaches its peak plasma concentration at approximately 24 h. In order to determine whether the effect of the A190 microemulsion in the CIPN model is mediated through activation of PPARα, we used a specific PPARα antagonist coupled with A190 treatment. The PPARα antagonist completely blocked the effect of the A190 microemulsion, suggesting that the effect of the drug is PPARα-dependent. The results from this study indicate that A190 microemulsion has the potential to increase the oral bioavailability of a highly lipophilic drug and partially reduce CIPN- and CFA-induced mechanical hypersensitivity. The histopathological analysis of key organs, including the liver, heart, spleen, kidney, and sections of the intestine (duodenum, jejunum, and ileum), following repeated oral administration of both vehicle microemulsion and A190 microemulsion in rats, revealed no signs of toxicity, indicating that the oral delivery of A190-loaded microemulsion is likely to be safe. PPARα agonists, including fibrates (e.g., fenofibrate), have been used for decades to manage dyslipidemia and are well tolerated, with an excellent safety profile. However, off-target side effects, including abnormal liver and kidney function biomarkers, have been reported.^60^ Further studies will investigate chronic exposure to assess potential off-target effects of the new PPARα agonist A190 and its microemulsion.

While this study highlights the potential of A190 microemulsion as a therapy for CIPN- and CFA-induced chronic inflammatory pain, certain limitations must be acknowledged: (1) we recognize the importance of long-term stability studies of A190 microemulsion to ensure its robustness and suitability for future clinical translation. (2) The multiple absorption peaks observed after oral administration of A190 microemulsion need further investigation. (3) The absence of a positive control group in the efficacy study. (4) A dose-dependent efficacy study of A190 microemulsion to determine the minimum effective dose. These limitations are recognized and will be addressed during future investigations.

## 5 Conclusions

This study identifies A190, a nonfibrate PPARα agonist, as a promising nonopioid therapeutic candidate for CIPN- and CFA-induced pain, conditions with no approved treatments. To overcome A190s poor solubility and permeability, a microemulsion formulation was developed and optimized, which achieved a 5-fold enhancement in oral bioavailability in vivo. Notably, oral administration of A190 microemulsion effectively alleviated both CIPN- and CFA-induced pain, highlighting the potential of microemulsion systems as effective delivery platforms for hydrophobic drugs and position A190 microemulsion as a safe and promising candidate for the treatment of chemotherapy-induced neuropathic pain and chronic inflammatory pain.

## Data Availability

All data needed to evaluate the conclusion are presented in the paper and/or the Supporting Information. The data that support the findings of this study are available from the corresponding author upon reasonable request.

## Supporting Information Available

Optimization, physicochemical characterization of vehicle, and A190 microemulsion; structure of A190; storage stability of A190 microemulsion, in vitro cytotoxicity of A190 microemulsion on HepG2 cells; and Caco-2 cells after incubation for 48 h (PDF)

## Notes

During the preparation of this work, the author(s) used ChatGPT to improve language since English is not the author’s first language. After using this tool/service, the author(s) reviewed and edited the content as needed and took full responsibility for the content of the publication.

## References

[ref1] Mezzanotte J. N.; Grimm M.; Shinde N. V.; Nolan T.; Worthen-Chaudhari L.; Williams N. O.; Lustberg M. B. Updates in the Treatment of Chemotherapy-Induced Peripheral Neuropathy. Curr. Treat. Options Oncol. 2022, 23 (1), 29–42. 10.1007/s11864-021-00926-0. 35167004 PMC9642075

[ref2] Desforges A. D.; Hebert C. M.; Spence A. L.; Reid B.; Dhaibar H. A.; Cruz-Topete D.; Cornett E. M.; Kaye A. D.; Urits I.; Viswanath O. Treatment and Diagnosis of Chemotherapy-Induced Peripheral Neuropathy: An Update. Biomed. Pharmacother. 2022, 147, 112671 10.1016/j.biopha.2022.112671. 35104697 PMC11118018

[ref3] Seretny M.; Currie G. L.; Sena E. S.; Ramnarine S.; Grant R.; MacLeod M. R.; Colvin L. A.; Fallon M. Incidence, Prevalence, and Predictors of Chemotherapy-Induced Peripheral Neuropathy: A Systematic Review and Meta-Analysis. Pain 2014, 155 (12), 2461–2470. 10.1016/j.pain.2014.09.020. 25261162

[ref4] Smith E. M. L.; Pang H.; Cirrincione C.; Fleishman S.; Paskett E. D.; Ahles T.; Bressler L. R.; Fadul C. E.; Knox C.; Le-Lindqwister N.; Gilman P. B.; Shapiro C. L.; et al. Effect of Duloxetine on Pain, Function, and Quality of Life Among Patients With Chemotherapy-Induced Painful Peripheral Neuropathy: A Randomized Clinical Trial. JAMA 2013, 309 (13), 1359 10.1001/jama.2013.2813. 23549581 PMC3912515

[ref5] Selvy M.; Cuménal M.; Kerckhove N.; Courteix C.; Busserolles J.; Balayssac D. The Safety of Medications Used to Treat Peripheral Neuropathic Pain, Part 1 (Antidepressants and Antiepileptics): Review of Double-Blind, Placebo-Controlled, Randomized Clinical Trials. Expet Opin. Drug Saf. 2020, 19 (6), 707–733. 10.1080/14740338.2020.1764934. 32363948

[ref6] Devchand P. R.; Keller H.; Peters J. M.; Vazquez M.; Gonzalez F. J.; Wahli W. The PPARα–leukotriene B4 pathway to inflammation control. Nature 1996, 384, 39–43. 10.1038/384039a0. 8900274

[ref7] Cho Y. R.; Lim J. H.; Kim M. Y.; Kim T. W.; Hong B. Y.; Kim Y.-S.; Chang Y. S.; Kim H. W.; Park C. W. Therapeutic Effects of Fenofibrate on Diabetic Peripheral Neuropathy by Improving Endothelial and Neural Survival in Db/Db Mice. PLoS One 2014, 9 (1), e83204 10.1371/journal.pone.0083204. 24392081 PMC3879243

[ref8] Esmaeili M. A.; Yadav S.; Gupta R. K.; Waggoner G. R.; Deloach A.; Calingasan N. Y.; Beal M. F.; Kiaei M. Preferential PPARα Activation Reduces Neuroinflammation, and Blocks Neurodegeneration in Vivo. Hum. Mol. Genet. 2016, 25 (2), 317–327. 10.1093/hmg/ddv477. 26604138 PMC4706116

[ref9] Griggs R. B.; Donahue R. R.; Adkins B. G.; Anderson K. L.; Thibault O.; Taylor B. K. Pioglitazone Inhibits the Development of Hyperalgesia and Sensitization of Spinal Nociresponsive Neurons in Type 2 Diabetes. J. Pain 2016, 17 (3), 359–373. 10.1016/j.jpain.2015.11.006. 26687453 PMC4791042

[ref10] Caillaud M.; Patel N. H.; Toma W.; White A.; Thompson D.; Mann J.; Tran T. H.; Roberts J. L.; Poklis J. L.; Bigbee J. W.; Fang X.; Gewirtz D. A.; Damaj M. I. A Fenofibrate Diet Prevents Paclitaxel-Induced Peripheral Neuropathy in Mice. Cancers 2021, 13 (1), 69 10.3390/cancers13010069. PMC7795224 33383736

[ref11] Huehnchen P.; Boehmerle W.; Endres M. Assessment of Paclitaxel Induced Sensory Polyneuropathy with “Catwalk” Automated Gait Analysis in Mice. PLoS One 2013, 8 (10), e76772 10.1371/journal.pone.0076772. 24143194 PMC3797113

[ref12] Caillaud M.; Patel N. H.; White A.; Wood M.; Contreras K. M.; Toma W.; Alkhlaif Y.; Roberts J. L.; Tran T. H.; Jackson A. B.; Poklis J.; Gewirtz D. A.; Damaj M. I. Targeting Peroxisome Proliferator-Activated Receptor-α (PPAR- α) to Reduce Paclitaxel-Induced Peripheral Neuropathy. Brain Behav. Immun. 2021, 93, 172–185. 10.1016/j.bbi.2021.01.004. 33434562 PMC8226373

[ref13] Li J.; Kennedy L. J.; Shi Y.; Tao S.; Ye X.-Y.; Chen S. Y.; Wang Y.; Hernández A. S.; Wang W.; Devasthale P. V.; Chen S.; Lai Z.; Zhang H.; Wu S.; Smirk R. A.; Bolton S. A.; Ryono D. E.; Zhang H.; Lim N.-K.; Chen B.-C.; Locke K. T.; O’Malley K. M.; Zhang L.; Srivastava R. A.; Miao B.; Meyers D. S.; Monshizadegan H.; Search D.; Grimm D.; Zhang R.; Harrity T.; Kunselman L. K.; Cap M.; Kadiyala P.; Hosagrahara V.; Zhang L.; Xu C.; Li Y.-X.; Muckelbauer J. K.; Chang C.; An Y.; Krystek S. R.; Blanar M. A.; Zahler R.; Mukherjee R.; Cheng P. T. W.; Tino J. A. Discovery of an Oxybenzylglycine Based Peroxisome Proliferator Activated Receptor α Selective Agonist 2-((3-((2-(4-Chlorophenyl)-5-Methyloxazol-4-Yl)Methoxy)Benzyl)(Methoxycarbonyl)Amino)Acetic Acid (BMS-687453). J. Med. Chem. 2010, 53 (7), 2854–2864. 10.1021/jm9016812. 20218621

[ref14] Oliveira A. C. P.; Bertollo C. M.; Rocha L. T. S.; Nascimento E. B.; Costa K. A.; Coelho M. M. Antinociceptive and Antiedematogenic Activities of Fenofibrate, an Agonist of PPAR Alpha, and Pioglitazone, an Agonist of PPAR Gamma. Eur. J. Pharmacol. 2007, 561 (1–3), 194–201. 10.1016/j.ejphar.2006.12.026. 17343847

[ref15] Blair H. A. Pemafibrate: First Global Approval. Drugs 2017, 77 (16), 1805–1810. 10.1007/s40265-017-0818-x. 28929345

[ref16] Dou X.; Nath D.; Shin H.; Nurmemmedov E.; Bourne P. C.; Ma J.-X.; Duerfeldt A. S. Evolution of a 4-Benzyloxy-Benzylamino Chemotype to Provide Efficacious, Potent, and Isoform Selective PPARα Agonists as Leads for Retinal Disorders. J. Med. Chem. 2020, 63 (6), 2854–2876. 10.1021/acs.jmedchem.9b01189. 32096640 PMC7365701

[ref17] Thanki K.; Gangwal R. P.; Sangamwar A. T.; Jain S. Oral Delivery of Anticancer Drugs: Challenges and Opportunities. J. Controlled Release 2013, 170 (1), 15–40. 10.1016/j.jconrel.2013.04.020. 23648832

[ref18] Poudel S.; Kim D. W. Developing PH-Modulated Spray Dried Amorphous Solid Dispersion of Candesartan Cilexetil with Enhanced In Vitro and In Vivo Performance. Pharmaceutics 2021, 13 (4), 497 10.3390/pharmaceutics13040497. 33917403 PMC8067465

[ref19] Narang A.; Delmarre D.; Gao D. Stable Drug Encapsulation in Micelles and Microemulsions. Int. J. Pharm. 2007, 345 (1–2), 9–25. 10.1016/j.ijpharm.2007.08.057. 17945446

[ref20] Yadav K. S.; Soni G.; Choudhary D.; Khanduri A.; Bhandari A.; Joshi G. Microemulsions for Enhancing Drug Delivery of Hydrophilic Drugs: Exploring Various Routes of Administration. Med. Drug Discovery 2023, 20, 100162 10.1016/j.medidd.2023.100162.

[ref21] Contreras K. M.; Caillaud M.; Neddenriep B.; Bagdas D.; Roberts J. L.; Ulker E.; White A. B.; Aboulhosn R.; Toma W.; Khalefa T.; Adel A.; Mann J. A.; Damaj M. I. Deficit in Voluntary Wheel Running in Chronic Inflammatory and Neuropathic Pain Models in Mice: Impact of Sex and Genotype. Behav. Brain Res. 2021, 399, 113009 10.1016/j.bbr.2020.113009. 33181181 PMC8961431

[ref22] Pangeni R.; Kang S.-W.; Oak M.; Park E. Y.; Park J. W. Oral Delivery of Quercetin in Oil-in-Water Nanoemulsion: In Vitro Characterization and in Vivo Anti-Obesity Efficacy in Mice. J. Funct. Foods 2017, 38, 571–581. 10.1016/j.jff.2017.09.059.

[ref23] Kumar R.; Sinha V. R. Preparation and Optimization of Voriconazole Microemulsion for Ocular Delivery. Colloids Surf., B 2014, 117, 82–88. 10.1016/j.colsurfb.2014.02.007. 24632034

[ref24] Koli A. R.; Ranch K. M.; Patel H. P.; Parikh R. K.; Shah D. O.; Maulvi F. A. Oral Bioavailability Improvement of Felodipine Using Tailored Microemulsion: Surface Science, Ex Vivo and in Vivo Studies. Int. J. Pharm. 2021, 596, 120202 10.1016/j.ijpharm.2021.120202. 33493600

[ref25] Pangeni R.; Jha S. K.; Maharjan R.; Choi J. U.; Chang K.-Y.; Choi Y. K.; Byun Y.; Park J. W. Intestinal Transport Mechanism and in Vivo Anticancer Efficacy of a Solid Oral Formulation Incorporating an Ion-Pairing Complex of Pemetrexed with Deoxycholic Acid Derivative. Int. J. Nanomed. 2019, 14, 6339–6356. 10.2147/IJN.S209722. PMC6690926 31496690

[ref26] Pangeni R.; Subedi L.; Jha S. K.; Kweon S.; Kang S.-H.; Chang K.-Y.; Choi J. U.; Byun Y.; Park J. W. Improvements in the Oral Absorption and Anticancer Efficacy of an Oxaliplatin-Loaded Solid Formulation: Pharmacokinetic Properties in Rats and Nonhuman Primates and the Effects of Oral Metronomic Dosing on Colorectal Cancer. Int. J. Nanomed. 2020, 15, 7719–7743. 10.2147/IJN.S267424. PMC7555381 33116497

[ref27] Kumar G.; Virmani T.; Pathak K.; Kamaly O. A.; Saleh A. Central Composite Design Implemented Azilsartan Medoxomil Loaded Nanoemulsion to Improve Its Aqueous Solubility and Intestinal Permeability: In Vitro and Ex Vivo Evaluation. Pharmaceuticals 2022, 15 (11), 1343 10.3390/ph15111343. 36355515 PMC9693424

[ref28] Toma W.; Kyte S. L.; Bagdas D.; Alkhlaif Y.; Alsharari S. D.; Lichtman A. H.; Chen Z.-J.; Del Fabbro E.; Bigbee J. W.; Gewirtz D. A.; Damaj M. I. Effects of Paclitaxel on the Development of Neuropathy and Affective Behaviors in the Mouse. Neuropharmacology 2017, 117, 305–315. 10.1016/j.neuropharm.2017.02.020. 28237807 PMC5489229

[ref29] Hartung J. E.; Eskew O.; Wong T.; Tchivileva I. E.; Oladosu F. A.; O’Buckley S. C.; Nackley A. G. Nuclear Factor-Kappa B Regulates Pain and COMT Expression in a Rodent Model of Inflammation. Brain. Behav. Immun. 2015, 50, 196–202. 10.1016/j.bbi.2015.07.014. 26187567 PMC4631655

[ref30] Chaplan S. R.; Bach F. W.; Pogrel J. W.; Chung J. M.; Yaksh T. L. Quantitative Assessment of Tactile Allodynia in the Rat Paw. J. Neurosci. Methods 1994, 53 (1), 55–63. 10.1016/0165-0270(94)90144-9. 7990513

[ref31] Hubatsch I.; Ragnarsson E. G. E.; Artursson P. Determination of Drug Permeability and Prediction of Drug Absorption in Caco-2 Monolayers. Nat. Protoc. 2007, 2 (9), 2111–2119. 10.1038/nprot.2007.303. 17853866

[ref32] Yu H.; Huang Q. Investigation of the Cytotoxicity of Food-Grade Nanoemulsions in Caco-2 Cell Monolayers and HepG2 Cells. Food Chem. 2013, 141 (1), 29–33. 10.1016/j.foodchem.2013.03.009. 23768322

[ref33] Manjusha V.; Rajeev M. R.; Anirudhan T. S. Magnetic Nanoparticle Embedded Chitosan-Based Polymeric Network for the Hydrophobic Drug Delivery of Paclitaxel. Int. J. Biol. Macromol. 2023, 235, 123900 10.1016/j.ijbiomac.2023.123900. 36870643

[ref34] Talegaonkar S.; Bhattacharyya A. Potential of Lipid Nanoparticles (SLNs and NLCs) in Enhancing Oral Bioavailability of Drugs with Poor Intestinal Permeability. AAPS PharmSciTech 2019, 20 (3), 121 10.1208/s12249-019-1337-8. 30805893

[ref35] Jha S. K.; Chung J. Y.; Pangeni R.; Choi H. S.; Subedi L.; Kweon S.; Choi J. U.; Byun Y.; Kim Y.-H.; Park J. W. Enhanced Antitumor Efficacy of Bile Acid-Lipid Complex-Anchored Docetaxel Nanoemulsion via Oral Metronomic Scheduling. J. Controlled Release 2020, 328, 368–394. 10.1016/j.jconrel.2020.08.067. 32890552

[ref36] Kim C.-K.; Cho Y.-J.; Gao Z.-G. Preparation and Evaluation of Biphenyl Dimethyl Dicarboxylate Microemulsions for Oral Delivery. J. Controlled Release 2001, 70 (1–2), 149–155. 10.1016/S0168-3659(00)00343-6. 11166415

[ref37] Keown P.; Niese D.; Cyclosporine Microemulsion Increases Drug Exposure and Reduces Acute Rejection without Incremental Toxicity in de Novo Renal Transplantation. Kidney Int. 1998, 54 (3), 938–944. 10.1046/j.1523-1755.1998.00042.x. 9734620

[ref38] Kim S. K.; Lee E. H.; Vaishali B.; Lee S.; Lee Y.; Kim C.-Y.; Moon H. T.; Byun Y. Tricaprylin Microemulsion for Oral Delivery of Low Molecular Weight Heparin Conjugates. J. Controlled Release 2005, 105 (1–2), 32–42. 10.1016/j.jconrel.2005.03.018. 15925422

[ref39] Fernández-Peña L.; Mojahid B. Z. E.; Guzmán E.; Ortega F.; Rubio R. G. Performance of Oleic Acid and Soybean Oil in the Preparation of Oil-in-Water Microemulsions for Encapsulating a Highly Hydrophobic Molecule. Colloids Interfaces 2021, 5 (4), 50 10.3390/colloids5040050.

[ref40] Borrajo M. L.; Quijano A.; Lapuhs P.; Rodriguez-Perez A. I.; Anthiya S.; Labandeira-Garcia J. L.; Valenzuela R.; Alonso M. J. Ionizable Nanoemulsions for RNA Delivery into the Central Nervous System – Importance of Diffusivity. J. Controlled Release 2024, 372, 295–303. 10.1016/j.jconrel.2024.06.051. 38909703

[ref41] Mittal P.; Vardhan H.; Ajmal G.; Bonde G. V.; Kapoor R.; Mittal A.; Mishra B. Formulation, Optimization, Hemocompatibility and Pharmacokinetic Evaluation of PLGA Nanoparticles Containing Paclitaxel. Drug Dev. Ind. Pharm. 2019, 45 (3), 365–378. 10.1080/03639045.2018.1542706. 30394795

[ref42] Rao J.; McClements D. J. Lemon Oil Solubilization in Mixed Surfactant Solutions: Rationalizing Microemulsion & Nanoemulsion Formation. Food Hydrocolloids 2012, 26 (1), 268–276. 10.1016/j.foodhyd.2011.06.002.

[ref43] Sarheed O.; Dibi M.; Ramesh K. V. R. N. S. Studies on the Effect of Oil and Surfactant on the Formation of Alginate-Based O/W Lidocaine Nanocarriers Using Nanoemulsion Template. Pharmaceutics 2020, 12 (12), 1223 10.3390/pharmaceutics12121223. 33348692 PMC7766092

[ref44] Fathordoobady F.; Sannikova N.; Guo Y.; Singh A.; Kitts D. D.; Pratap-Singh A. Comparing Microfluidics and Ultrasonication as Formulation Methods for Developing Hempseed Oil Nanoemulsions for Oral Delivery Applications. Sci. Rep. 2021, 11 (1), 72 10.1038/s41598-020-79161-w. 33420136 PMC7794282

[ref45] Dalvi S. V.; Dave R. N. Controlling Particle Size of a Poorly Water-Soluble Drug Using Ultrasound and Stabilizers in Antisolvent Precipitation. Ind. Eng. Chem. Res. 2009, 48 (16), 7581–7593. 10.1021/ie900248f.

[ref46] Qian C.; McClements D. J. Formation of Nanoemulsions Stabilized by Model Food-Grade Emulsifiers Using High-Pressure Homogenization: Factors Affecting Particle Size. Food Hydrocolloids 2011, 25 (5), 1000–1008. 10.1016/j.foodhyd.2010.09.017.

[ref47] Hecht L. L.; Wagner C.; Landfester K.; Schuchmann H. P. Surfactant Concentration Regime in Miniemulsion Polymerization for the Formation of MMA Nanodroplets by High-Pressure Homogenization. Langmuir 2011, 27 (6), 2279–2285. 10.1021/la104480s. 21314152 PMC3052788

[ref48] Hassan S. F.; Asghar S.; Ullah Khan I.; Munir R.; Khalid S. H. Curcumin Encapsulation in Geranium Oil Microemulsion Elevates Its Antibacterial, Antioxidant, Anti-Inflammatory, and Anticancer Activities. ACS Omega 2024, 9 (5), 5624–5636. 10.1021/acsomega.3c08033. 38343911 PMC10851249

[ref49] Suhail N.; Alzahrani A. K.; Basha W. J.; Kizilbash N.; Zaidi A.; Ambreen J.; Khachfe H. M. Microemulsions: Unique Properties, Pharmacological Applications, and Targeted Drug Delivery. Front. Nanotechnol. 2021, 3, 754889 10.3389/fnano.2021.754889.

[ref50] Sharma N.; Kaur G.; Khatkar S. K. Optimization of Emulsification Conditions for Designing Ultrasound Assisted Curcumin Loaded Nanoemulsion: Characterization, Antioxidant Assay and Release Kinetics. LWT 2021, 141, 110962 10.1016/j.lwt.2021.110962.

[ref51] Wik J.; Bansal K. K.; Assmuth T.; Rosling A.; Rosenholm J. M. Facile Methodology of Nanoemulsion Preparation Using Oily Polymer for the Delivery of Poorly Soluble Drugs. Drug Delivery Transl. Res. 2020, 10 (5), 1228–1240. 10.1007/s13346-019-00703-5. PMC7447668 31858441

[ref52] Kapate N.; Clegg J. R.; Mitragotri S. Non-Spherical Micro- and Nanoparticles for Drug Delivery: Progress over 15 Years. Adv. Drug Delivery Rev. 2021, 177, 113807 10.1016/j.addr.2021.05.017. 34023331

[ref53] Subramanian P.; Siddalingam R. Self-Nanoemulsifying Drug Delivery Systems of Poorly Soluble Drug Dutasteride: Formulation and In-Vitro Characterization. J. Appl. Pharm. Sci. 2017, 4, 11 10.7324/JAPS.2017.70402.

[ref54] Shi Y.; Zhang M.; Chen K.; Wang M. Nano-Emulsion Prepared by High Pressure Homogenization Method as a Good Carrier for Sichuan Pepper Essential Oil: Preparation, Stability, and Bioactivity. LWT 2022, 154, 112779 10.1016/j.lwt.2021.112779.

[ref55] Zheng Y.; Zhao C.; Chen B.; Teng H.; Ai C.; Chen L. D-α-Tocopherol Polyethylene Glycol 1000 Succinate-Based Microemulsion Delivery System: Stability Enhancement of Physicochemical Properties of Luteolin. Food Chem. 2023, 426, 136587 10.1016/j.foodchem.2023.136587. 37364422

[ref56] Gharbavi M.; Manjili H. K.; Amani J.; Sharafi A.; Danafar H. In Vivo and in Vitro Biocompatibility Study of Novel Microemulsion Hybridized with Bovine Serum Albumin as Nanocarrier for Drug Delivery. Heliyon 2019, 5 (6), e01858 10.1016/j.heliyon.2019.e01858. 31198875 PMC6556858

[ref57] Vyas T. K.; Shahiwala A.; Amiji M. M. Improved Oral Bioavailability and Brain Transport of Saquinavir upon Administration in Novel Nanoemulsion Formulations. Int. J. Pharm. 2008, 347 (1–2), 93–101. 10.1016/j.ijpharm.2007.06.016. 17651927 PMC2213794

[ref58] Dahan A.; Hoffman A. The Effect of Different Lipid Based Formulations on the Oral Absorption of Lipophilic Drugs: The Ability of in Vitro Lipolysis and Consecutive Ex Vivo Intestinal Permeability Data to Predict in Vivo Bioavailability in Rats. Eur. J. Pharm. Biopharm. 2007, 67 (1), 96–105. 10.1016/j.ejpb.2007.01.017. 17329087

[ref59] Scripture C.; Figg W.; Sparreboom A. Peripheral Neuropathy Induced by Paclitaxel: Recent Insights and Future Perspectives. Curr. Neuropharmacol. 2006, 4 (2), 165–172. 10.2174/157015906776359568. 18615126 PMC2430667

[ref60] Yamashita S.; Masuda D.; Matsuzawa Y. Pemafibrate a New Selective PPARα Modulator: Drug Concept and Its Clinical Applications for Dyslipidemia and Metabolic Diseases. Curr. Atherosclerosis Rep. 2020, 22 (1), 5 10.1007/s11883-020-0823-5. PMC6978439 31974794

