# Supplementary material for: New PPARα Agonist A190-Loaded Microemulsion for Chemotherapy-Induced Peripheral Neuropathy
Source: Mol Pharm. 2025 Jan 29;22(3):1641–56. doi: 10.1021/acs.molpharmaceut.4c01374 (PMC11881135; doi:10.1021/acs.molpharmaceut.4c01374)
Supplement: Supplementary file 1 — mp4c01374_si_001.pdf [file mp4c01374_si_001.pdf]

Supplementary Materials

New PPARα agonist A190 loaded microemulsion for chemotherapy-induced peripheral neuropathy

Rudra Pangeni [1,#], Surendra Poudel [1,#], Sara M. Herz [2], Grant Berkbigler [3], Adam S. Duerfeldt [3*], M. Imad Damaj [2*], Qingguo Xu [1,4*]

[1]Department of Pharmaceutics, School of Pharmacy, Virginia Commonwealth University, Richmond, VA 23298, USA

[2]Department of Pharmacology and Toxicology, School of Medicine, Virginia Commonwealth University, Richmond, VA 23298, USA

[3]Department of Medicinal Chemistry, College of Pharmacy, University of Minnesota, Minneapolis, MN 55455, USA

[4]Departments of Ophthalmology, Pediatrics, Biomedical Engineering, and Massey Cancer Center, Center for Pharmaceutical Engineering, and Center for Drug Discovery, Virginia Commonwealth University, Richmond, VA 23298, USA

Table S1. Statistical ANOVA results of BBD for optimization of mean droplet size, PDI, zeta potential, drug content of A190 microemulsion.

Source	Mean droplet size (Y_1)		PDI (Y_2)		Zeta potential (Y_3)		Drug content (Y_4)	
	F-value	P-value	F-value	P-value	F-value	P-value	F-value	P-value
**Model**	7.97	<0.05*	3.36	<0.05*	1.45	0.36	4.68	<0.05*
X_1	48.70	<0.01**	14.90	<0.05*	<0.01	0.94	25.70	<0.01**
X_2	3.52	0.12	0.04	0.85	<0.01	0.95	1.32	0.30
X_3	8.25	<0.05*	1.97	0.22	1.61	0.26	0.14	0.72
X_1X_2	1.46	0.28	0.04	0.85	0.06	0.82	<0.01	0.98
X_1X_3	2.05	0.21	0.76	0.42	0.70	0.44	1.73	0.25
X_2X_3	<0.01	0.97	0.02	0.90	0.23	0.65	0.04	0.85
X_1^2	2.50	0.17	0.29	0.61	3.13	0.14	9.80	0.03
X_2^2	0.86	0.40	1.35	0.30	1.45	0.28	1.73	0.25
X_3^2	4.25	0.09	11.60	<0.05*	5.26	0.07	1.36	0.30
R^2	0.950		0.984		0.957		0.957	
**Lack of fit**	0.20	0.89	5.32	0.16	3.63	0.22	1.01	0.53

Notes: The significance of the effect of independent variables on responses: *P<0.05; **P<0.01

Table S2. Mean droplet size, PDI, and zeta potential of vehicle microemulsion and A190 microemulsion.

Sample	Droplet size (nm)	PDI	Zetapotential (mV)
A190 microemulsion	149.9 ± 1.58	0.136 ± 0.030	-8.51 ± 0.33
Vehicle microemulsion	119.8 ± 1.64	0.131 ±0.007	-5.92 ± 0.87

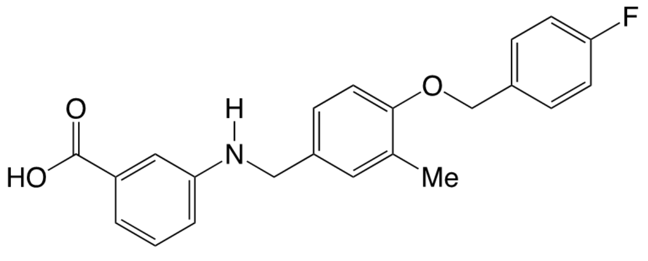

Figure S1. Chemical structure of A190 (Reprinted with permission from [16]. Copyright ©2020, American Chemical Society).

(16) Dou, X.; Nath, D.; Shin, H.; Nurmemmedov, E.; Bourne, P. C.; Ma, J.-X.; Duerfeldt, A. S. Evolution of a 4-Benzyloxy-Benzylamino Chemotype to Provide Efficacious, Potent, and Isoform Selective PPARα Agonists as Leads for Retinal Disorders. *J. Med. Chem.* **2020**, *63* (6), 2854–2876.

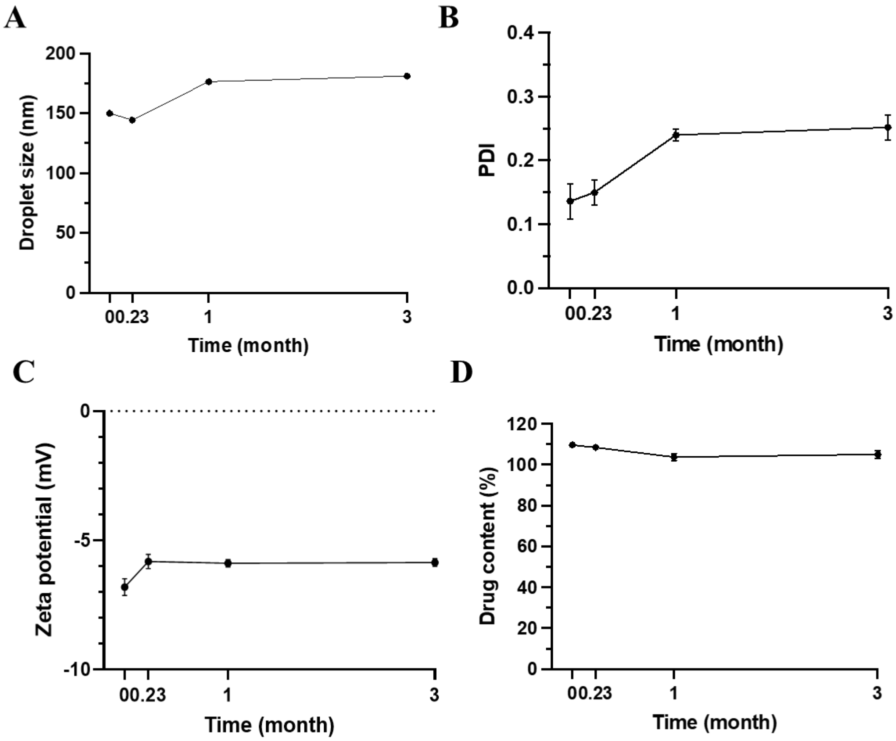

Figure S2. Storage stability of A190 microemulsion at room temperature (25 ± 5°C). **(A)** Average droplet size, **(B)** Polydispersity index, **(C)** Zeta potential, and **(D)** Drug content of A190 microemulsion (mean ± SD; n = 3).

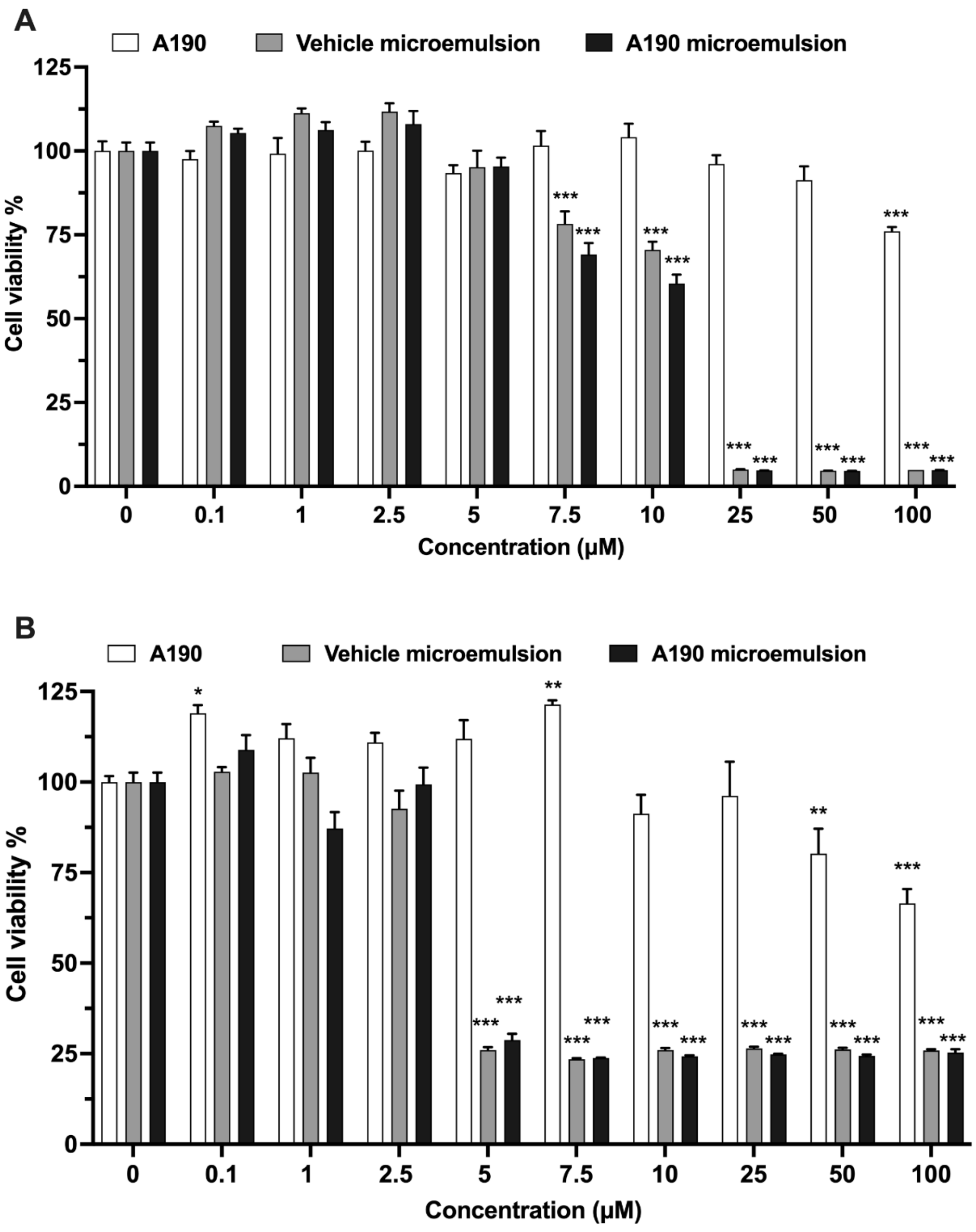

Figure S3. In vitro cytotoxic effects of A190 in 0.2% dimethyl sulfoxide (DMSO), vehicle microemulsion, and A190 microemulsion on (A) HepG2 cells and (B) Caco-2 cells after incubation for 48 h. Values are mean ± SD (n=5). $^{*}p < 0.05$, $^{**}p < 0.01$, $^{***}p < 0.001$ compared to respective DMEM or 0.2% DMSO controls.
